# Tapeworm infection affects sleep-like behavior in three-spined sticklebacks

**DOI:** 10.1038/s41598-024-73992-7

**Published:** 2024-10-08

**Authors:** Marc B. Bauhus, Sina Mews, Joachim Kurtz, Alexander Brinker, Robert Peuß, Jaime M. Anaya-Rojas

**Affiliations:** 1 Institute for Evolution and Biodiversity, University of Münster, Hüfferstraße 1, 48149 Münster, Germany; 2 Department of Business Administration and Economics, Bielefeld University, Universitätsstraße 25, 33614 Bielefeld, Germany; 3 Fisheries Research Station Baden-Württemberg, Argenweg 50/1, 88085 Langenargen, Germany; 4 Institute for Limnology, University of Constance, Mainaustraße 252, 78464 Constance, Germany; 5 Joint Institute for Individualisation in a Changing Environment, University of Münster and Bielefeld University, Münster, Bielefeld, Germany

**Keywords:** Host-parasite interaction, Sleep behavior, *Gasterosteus aculeatus*, *Schistocephalus solidus*, Infection, Animal behaviour, Animal physiology

## Abstract

Sleep is a complex and conserved biological process that affects several body functions and behaviors. Evidence suggests that there is a reciprocal interaction between sleep and immunity. For instance, fragmented sleep can increase the probability of parasitic infections and reduce the ability to fight infections. Moreover, viral and bacterial infections alter the sleep patterns of infected individuals. However, the effects of macro-parasitic infections on sleep remain largely unknown, and measuring sleep in non-model organisms remains challenging. In this study, we investigated whether macro-parasite infections could alter sleep-like behavior of their hosts. We experimentally infected three-spined sticklebacks (*Gasterosteus aculeatus*), a freshwater fish, with the tapeworm *Schistocephalus solidus* and used a hidden Markov model to characterize sleep-like behavior in sticklebacks. One to four days after parasite exposure, infected fish showed no difference in sleep-like behavior compared with non-exposed fish, and fish that were exposed-but-not-infected only showed a slight reduction in sleep-like behavior during daytime. Twenty-nine to 32 days after exposure, infected fish showed more sleep-like behavior than control fish, while exposed-but-not-infected fish showed overall less sleep-like behavior. Using brain transcriptomics, we identified immune- and sleep-associated genes that potentially underlie the observed behavioral changes. These results provide insights into the complex association between macro-parasite infection, immunity, and sleep in fish and may thus contribute to a better understanding of reciprocal interactions between sleep and immunity.

**Supplementary Information:**

The online version contains supplementary material available at 10.1038/s41598-024-73992-7.

## Introduction

Sleep is a fundamental biological process in animals^1^ and is characterized by periods of behavioral inactivity that are easily reversible^2,3^. Current hypotheses assume that sleep has evolved to maintain brain connectivity, plasticity, and health, and to reduce caloric consumption^4,5^. It is regulated by neurological processes^6^ circadian rhythms, and duration of wakefulness^7^.

Furthermore, parasitic infections and resulting immune responses can affect sleep regulation of the host, either by increasing^8,9^ or decreasing^10,11^ duration and intensity of sleep^8–11^ in association with infection intensity and timepoint after exposure^12,13^. These infection effects on sleep are induced by inflammatory cytokines^11,12,14,15^, which are thought to interact with sleep-regulating neuropeptides in the brain. For example, the wakefulness-promoting neuropeptide hypocretin/orexin influences the cellular composition of the immune system via cytokine-mediated signaling upon sleep deprivation^16^. Although the impact of bacterial and viral infections on sleep-immunity interactions has been extensively studied in mammals^8,9,13,17^, the influence of macro-parasite infections on such interactions remains largely unexplored. Furthermore, substantial knowledge is still lacking when and why sleep is altered in the course of the infection. The persisting and long-term infection dynamics of a macro-parasite infection might help to better understand the differential infection effects on sleep.

**Fig. 1:**
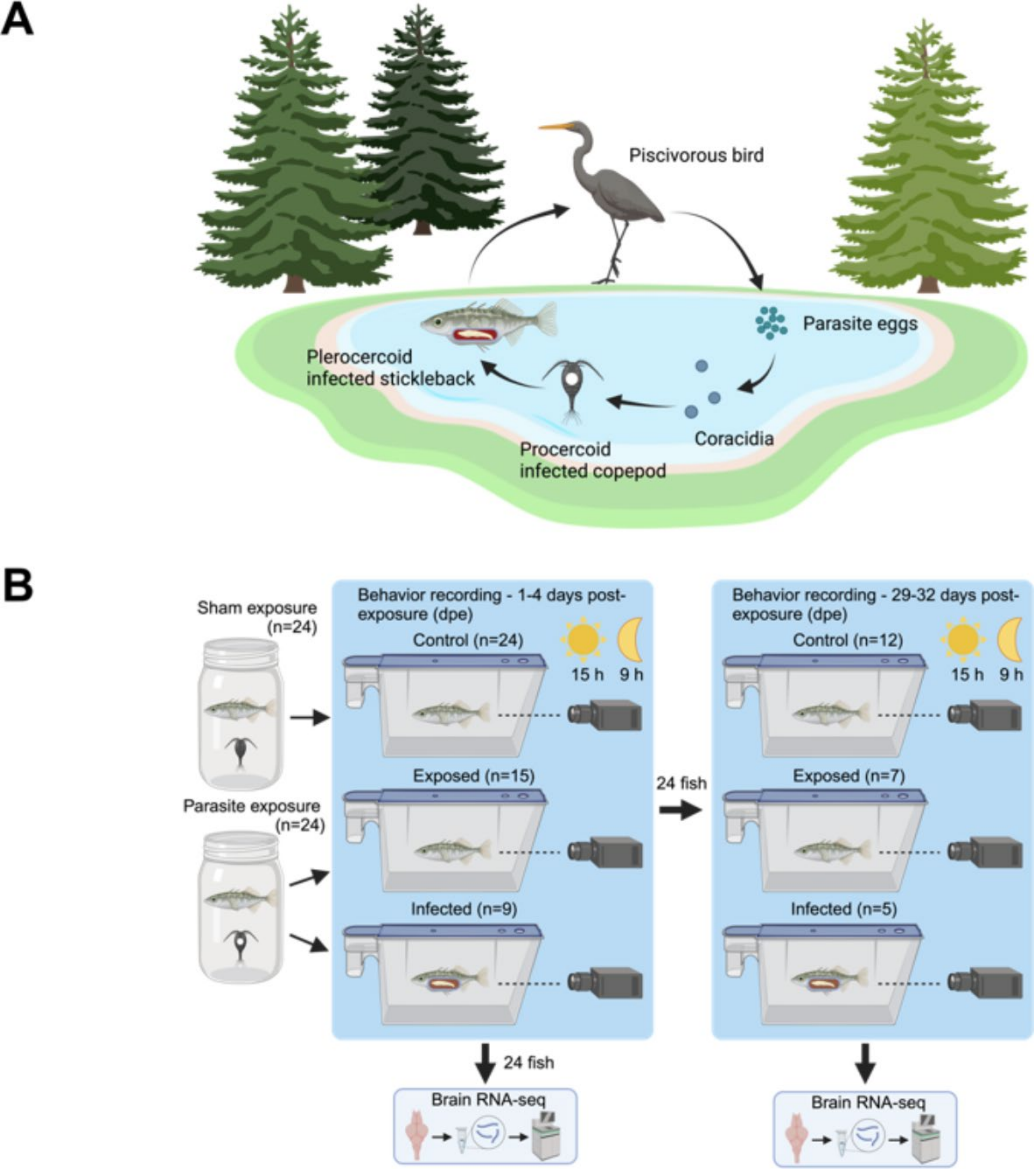
Tracking locomotor activity of sticklebacks to estimate sleep upon infection with *S. solidus*. (**A**) Lifecycle of *S. solidus*, a trophically transmitted tapeworm with two intermediate hosts—cyclopoid copepods and three-spined sticklebacks—and warm-blooded fish-eating animals (mostly birds) as final hosts, where the worms reproduce^18^. Created in BioRender.^84^ (**B**) Experimental design. Individual sticklebacks were exposed to either parasite-naive copepods or *S. solidus*-infected copepods (indicated by white dots). The resulting non-exposed (control), exposed but not infected (exposed), and infected (infected) fish were video recorded 1–4 and 29–32 days post parasite exposure (dpe). Half of the fish were dissected after the first recording, whereas the other half were recorded at both time points and then dissected. All fish were inspected for their infection status, and brain tissue was used for RNA-seq, providing transcriptomic data for the time points 4 and 32 dpe. Created in BioRender.^85^

In this study, we aimed to address these gaps by examining the effects of a macro-parasite infection on sleep in a freshwater fish host. For this, we made use of the three-spined stickleback (*Gasterosteus aculeatus*) and its tapeworm *Schistocephalus solidus*^18–22^ (Fig. 1A). We experimentally infected sticklebacks and measured the effects of the infection on the overall activity of individuals during two four-day periods, 1–4 days and 29–32 days after parasite exposure (dpe) (Fig. 1B). *Schistocephalus solidus* is a trophically transmitted parasite with a three-host life cycle, i.e. cyclopoid copepods and sticklebacks as intermediate host and fish-eating birds as the final host^18^ (Fig. 1A). In sticklebacks, *S. solidus* migrates through the gut wall^23^ and grows dramatically in the body cavity, where it manipulates the host’s behavior towards showing less anti-predator behaviors to increase the chances of transmission to the final host^24,25^. In general, sticklebacks show periods of activity and inactivity when exposed to light-dark conditions that resemble sleep. However, individuals vary strongly in their daily activity patterns, and while some individuals are mostly nocturnal, others are active around the clock^26^. Thanks to the increasing understanding of *S. solidus* biology and its effects on stickleback behavior and immunity, this host-parasite system is well-suited to experimentally study the effects of macro-parasitic infections on sleep behavior in vertebrate hosts.

**Fig. 2:**
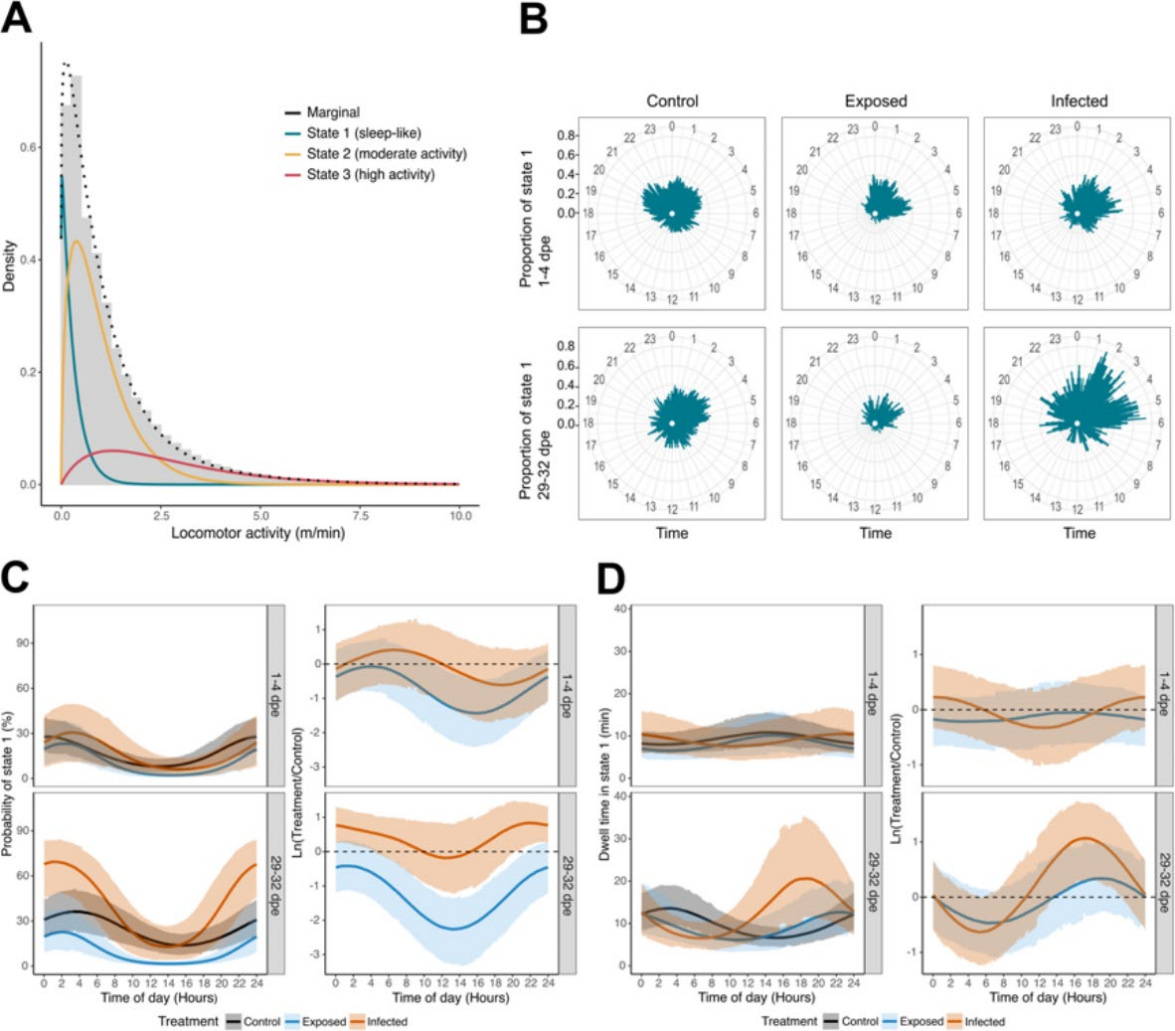
Stickleback sleep increased upon *S. solidus* infection 29–32 days post parasite exposure. (**A**) Histogram of three different behavioral states identified by the hidden Markov model fitted to the observed locomotor activity. The state-dependent distributions were weighted according to the proportion of time spent in different states. The dashed line indicates the associated marginal distribution under the fitted model. (**B**) Mean proportions of time spent in state 1 in control, exposed, and infected fish averaged over 24 h 1–4 and 29–32 dpe. (**C**) Left side: Probabilities for control, exposed, and infected fish occupying state 1, corresponding to the periodic stationary distribution of the HMM. Middle lines display the mean probabilities. Upper and lower areas represent the respective 95% confidence intervals (CIs). Right side: Logarithmic ratio of the deviation of exposed and infected fish from the control (dashed line) was derived from simulations based on the HMM. Middle lines display the means. Upper and lower areas represent the respective 95% CIs. (**D**) Left side: Expected dwell times (i.e., time spent continuously in one state) as a function of time of day for control, exposed, and infected fish in state 1. Middle lines display the mean dwell times. Upper and lower lines represent the respective 95% CIs. Right side: Logarithmic ratio of the deviation of exposed and infected fish from the control (dashed line) derived from simulations based on the HMM. Middle lines display the means. Upper and lower areas represent the respective 95% CIs.

However, defining and measuring sleep without the use of electroencephalograms is very challenging, particularly in aquatic organisms. Hence, one must rely on observed behavioral patterns and in most cases subjective decisions to detect and define sleep-like behaviors^3,27–29^. To overcome these challenges, we made use of hidden Markov models (HMMs) and objectively identified three distinct behavioral states in our experimental fish: a low activity and sleep-like behavioral state, a state of moderate activity, and a state of high activity (Fig. 2A). We compared the proportion and probability of sleep-like behavior between three types of experimental fish: control (fish that were not exposed to *S. solidus*), exposed (fish that were exposed but not infected), and infected (Fig. 1B). We predicted that during the early stage of infection (1–4 dpe), both infected and exposed fish would show less sleep-like behavior than control fish, because a successful clearance of the parasite possibly caused by an acute inflammatory response might take place^30^ and could lead to sleep disruptions^10,11,13^. We further predicted that at a later stage of infection (29–32 dpe), infected fish might show more sleep-like behavior due to the energetic demand of the parasite, thereby increasing the need for more efficient energy conservation and allocation to fight the infection^31^. Moreover, the immune system might still be activated even though it is unable of effectively clearing the parasite at this infection stage, which could have sleep-promoting effects^8,9^. An alternative prediction would be that infected fish also show less sleep-like behavior at this stage, if the infection causes chronic inflammation^32–34^. In addition to the estimation of sleep-like behavior, we sequenced the transcriptome of fish brains at four and 32 dpe to identify potential mediators of the sleep-immunity interactions triggered by *S. solidus* infection.

## Results and discussion

Overall, we found that sticklebacks spent approximately 19% of their time in a sleep-like behavioral state, mostly during the night (i.e., between 21:30 and 6:30, Fig. 2B and Supplementary Fig. 2). We found partial support for our predictions. In the early stages of infection (1–4 dpe), we observed weak differences in sleep-like behavior among the three types of fish (Fig. 2B to D and Supplementary Fig. 2).

**Fig. 3:**
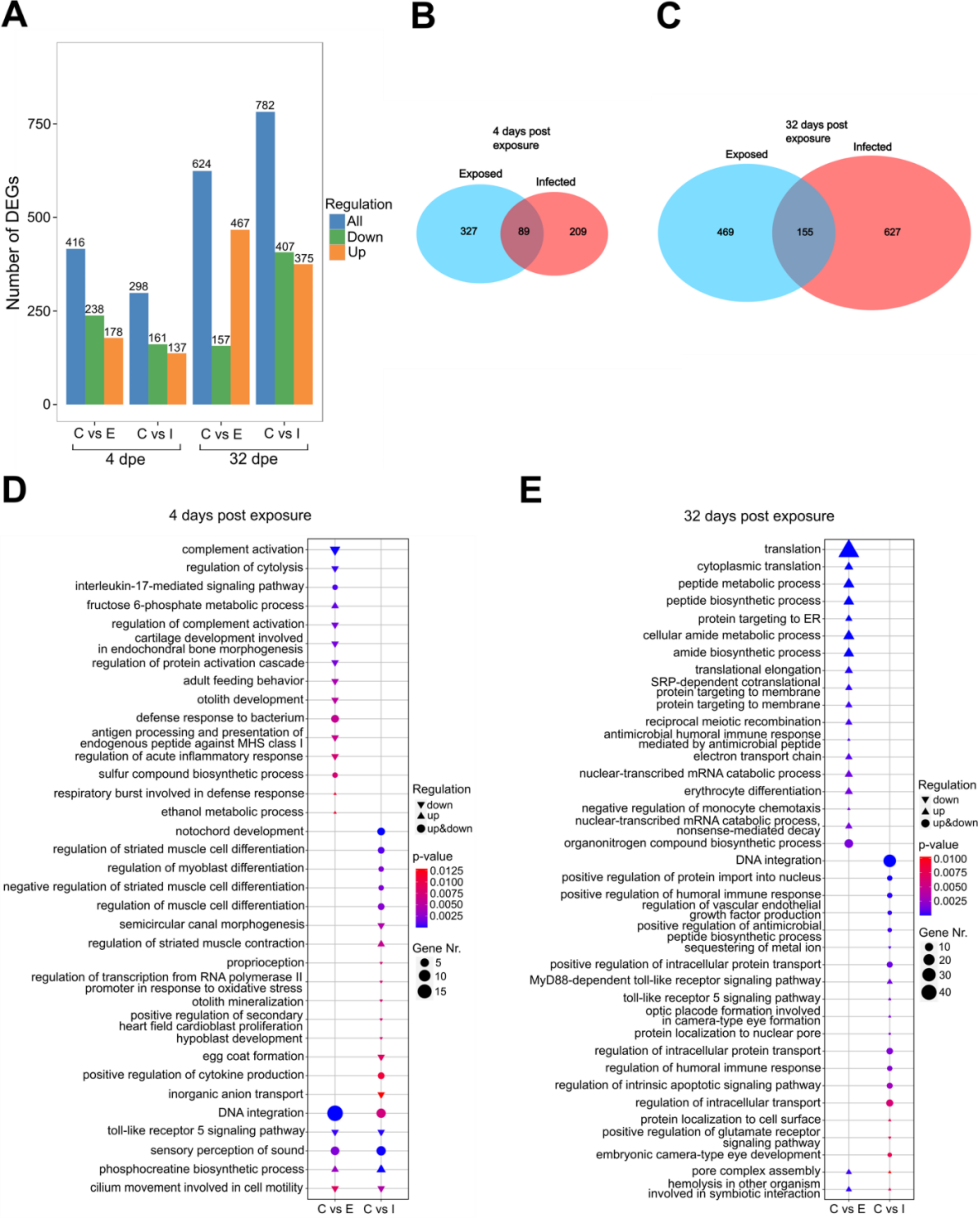
Differential gene expression patterns and GO-terms between control, exposed, and infected fish. (**A**) Number of differentially expressed genes (DEGs) (total, up-, and downregulated) of the control (C) versus exposed (E) and infected (I) fish at 4 and 32 dpe. (**B**) Venn diagram showing the number of unique and shared DEGs between exposed and infected fish versus control fish at 4 dpe (**C**) and 32 dpe. (**D**) The 20 most significantly enriched and non-redundant Gene Ontology (GO) terms between control and exposed but not infected and infected fish at 4 dpe (**E**) and 32 dpe. The shape indicates the direction of the regulation. The size of the shape shows the number of DEGs expressed in this GO term and the color gradient represents the significance of enrichment.

These results suggest little impact of the tapeworm infection on sleep-like behavior during the first four days of infection, perhaps because *S. solidus* can effectively evade the immune response of sticklebacks^30,35^. This hypothesis is supported by our brain transcriptome analysis, which shows that there was only one immune response-related GO-term among the most significantly enriched GO-terms (‘positive regulation of cytokine production’, Fig. 3D). However, some immune-related genes were differentially expressed between infected and control fish. For example, the colony stimulating factor 1 receptor a (CSF1Ra), which is involved in neuroinflammation and monocyte (microglia) differentiation and proliferation – key processes for maintaining brain homeostasis and health^36^—was upregulated in infected fish at four days post exposure (Table 1).

**Table 1:**
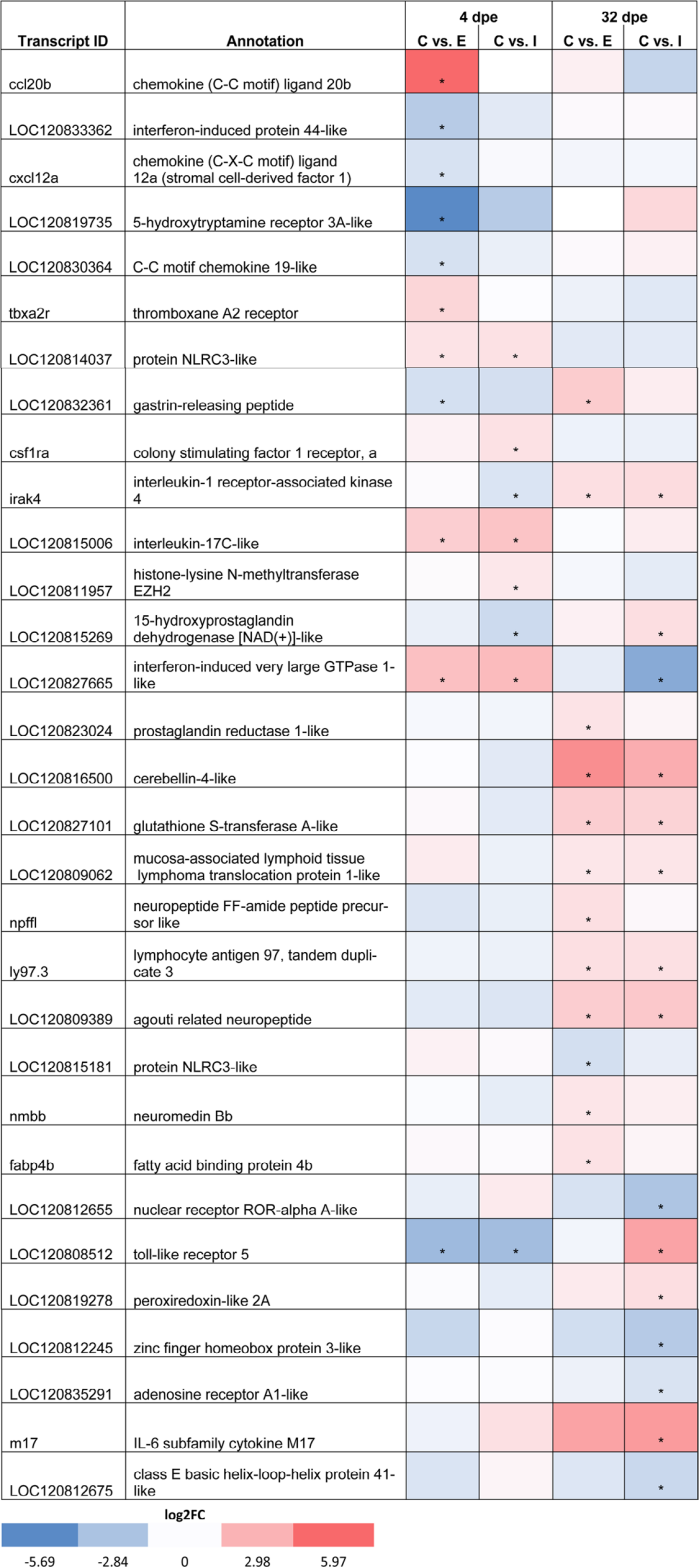
Immune- and sleep-associated genes are differentially expressed in exposed and infected fish.

Table containing the NCBI transcript ID, annotation and heat map showing the log2 fold change of each differentially expressed gene per treatment (Control = C, Exposed = E, Infected = I) and time point (4 and 32 dpe). Significant up- or down-regulation is indicated with an asterisk (log2FC > 1, p </= 0.05). For a short summary of the (putative) biological function for each gene see Supplementary table 3.

Furthermore, we found that exposed-but-not-infected fish had a slightly lower probability of being in a sleep-like state during the daytime (6:30–21:30) than control fish at 1–4 dpe (approximately 7%, 95% CI: − 18.6 to 2.6%; Fig. 2C). The brain transcriptomic analysis revealed that in the exposed-but-not-infected fish the chemokine (C–C motif) ligand 20b (ccl20b) was upregulated compared to control fish (Table 1). As ccl20b is involved in neuroinflammation, chemokine binding, cytokine response, and leukocyte chemotaxis^37,38^, the observed upregulation of this gene may indicate an immune response against the parasite or translocated gut bacteria following gut perforation by the parasite^39^. However, whether these or other genes involved in neuroinflammation influenced these small changes in the propensity of exposed-but-not-infected fish towards less sleep-like behavior still needs to be functionally validated. Moreover, exposed-but-not-infected fish may also result from individual parasites which failed to infect the fish^40^, in which case we might not expect any immune responses^40^.

As predicted for the later stage of infection (29–32 dpe), we found that infected fish showed more sleep-like behavior than control fish (Fig. 2B,C), especially during the night hours (21:30–06:30). Overall, the probability of sleep-like behavior during the night for infected fish increased from approximately 26.4% (95% CI: 7.3 to 46.7%) to 62.5% (95% CI: 39.1 to 82.2%); during the day, it increased from 12% (95% CI: 1.5 to 28.9%) to 26.0% (95% CI: 4.8 to 56.1%; Fig. 2C). Specifically, during the night, the probability of sleep-like behavior for infected fish was approximately 30% higher than that of control fish (95% CI: 3.6 to 54.9% of sleep) and 43% higher than that of exposed-but-not-infected fish (95% CI: 17.6 to 65.9% of sleep-like behavior). Furthermore, the time that infected fish spent uninterruptedly in a sleep-like state during the day (i.e., the expected time spent in state 1 before switching to a different state) was longer than that at night. During the nights, infected fish spent approximately 10 min uninterruptedly in a sleep-like state (95% CI: 5.7 to 15.4 min) 1–4 dpe and 11.5 min (95% CI: 4.3 to 21.4 min) 29–32 dpe. However, during the day, the differences in the time spent uninterruptedly in a sleep-like state changed from approximately 8.9 min (95% CI: 4.5 to 14.1 min) to 14.07 min (95% CI: 3.9 to 21.1 min) from the early (1–4 dpe) to the later (29–32 dpe) infection state (Fig. 2D). Interestingly, during the second part of the day (i.e., from 12:00 to 21:00) infected fish spent almost 10 min more uninterruptedly in a sleep-like state than control fish (95% CI: − 0.8 to 24.3 min) and approximately 8.8 min more than exposed-but-not-infected fish (95% CI: − 3.0 to 22.7 min; Fig. 2D).

Within the first twelve weeks of infection, *S. solidus* shows a steep increase in weight gain^41^. If the energetic cost of *S. solidus* is high, infected fish require more sleep to compensate for their energetic requirements. There is ample evidence to support this hypothesis. For example, *S. solidus* drains a significant amount of energy from its stickleback host, particularly during single infections^42^. Additionally, when infected with large intestinal parasites, fish often specialize in eating many smaller prey items or selectively preying on a few larger items to compensate for the energetic cost of infection^43^. Hence, infected sticklebacks could cope with the cost of infection by sleeping more^31^.

The increased sleep-like behavior of infected fish at 29–32 dpe could also be partially explained by the direct and indirect effects of the immune evasion strategies of *S. solidus*. Immune evasion is a key strategy used by pathogens to improve their fitness^44^. For trophically transmitted parasites, such as *S. solidus*, the energy invested in immune evasion has to trade-off with somatic growth; therefore, the increased sleep observed in the later infection stage could be a way to modulate the host immune system to enhance nutrient gain for the parasite^44^.

Immune responses against bacterial or viral infections, as well as the experimental inoculation of inflammatory cytokines, can have sleep-promoting effects, depending on the infection dose and stage^9,12,13,45–48^. This is thought to contribute to a more efficient defense against parasites^49,50^. Therefore, this might also be true for long-term infections with macro-parasites such as tapeworms. Although a successful clearance of the tapeworm is unlikely to occur late during infection, because the parasite usually evades an effective immune response, the immune system might still be active to reduce parasite growth^30^. We found that both infected and exposed fish had more differentially expressed genes (DEGs) than the control fish at the later time point (32 dpe) compared to the early (4 dpe) time point (Fig. 3A). Among these DEGs, we identified several genes with possible associations with immune response and sleep regulation (Table 1). For example, the anti-inflammatory and circadian rhythm-associated nuclear receptor ROR-alpha A-like (RORA A-like)^51^ was downregulated in infected fish, whereas several inflammation-associated genes such as IL-6 subfamily cytokine M17 were upregulated (Table 1). IL-6 enhances sleep in rats^52^ and in moderate concentrations also in humans^12^. A high functional redundancy within the IL-6 family^53^ suggests that M17 could have similar effects on sleep even though this still needs to be functionally validated. Therefore, these upregulated inflammation-associated genes may have contributed to the observed increase in sleep-like behavior during night hours.

*Schistocephalus solidus*, such as many other trophically transmitted parasites with complex life cycles^54–56^, is known to manipulate the anti-predatory behavior of its stickleback host to increase the likelihood of transmission to the final host^24^. Our results suggest that parasites might make use of immune-sleep interactions in their host to increase the arousal threshold of infected individuals during sleep to increase the predation risk of the stickleback^4,24^. In fact, we observed that infected fish spent on average more time continuously in a sleep-like state (dwell time) during the daytime (Fig. 2D), when the predation risk by birds is thought to be highest^57,58^. In a recent brain transcriptomics study using stickleback after three month of infection with *S. solidus*, it was suggested that the inositol pathway which is involved in human neuropsychiatric diseases^59^ might be engaged in the behavioral manipulation by the parasite^60^. However, we did not find a significant differential expression of the associated gene inositol monophosphatase 1 (impa1) at 1–4 or 29–32 dpe. It is likely that the parasites in our experiment were not yet infective to the final host, because they were below the published threshold mass for reproduction in the final host of ~ 50 mg^24^ (Supplementary Table 1). A study in the copepod intermediate host of *S. solidus* even showed a reduced predation of infected copepods by sticklebacks before the parasite becomes infective to the stickleback host^61^, which might also take place in infected sticklebacks. Nevertheless, it is possible that the 50 mg mass threshold is lower for some parasite populations or that the behavioral manipulation starts with changes in sleep and extends to other behaviors that increase predation risk after reaching the 50 mg mass threshold. Therefore, further experiments at later stages of infection are needed to test whether the fish show more sleep-like behavior than in our experiment.

We also found that exposed fish showed an overall lower proportion of sleep-like behavior (Fig. 2B,C), and also a lower probability of showing this behavior during the daytime (Fig. 2D) compared to control fish 29–32 dpe. Interestingly, these fish also showed a higher number of upregulated GO terms and DEGs associated with translation and protein transport (Fig. 3E). We also identified upregulated genes that were directly related to neuroinflammation, neuropeptide activity, and sleep regulation (Table 1). For example, interleukin-1 receptor-associated kinase 4 (irak4) facilitates the inflammatory response^62^, and agouti-related neuropeptide promotes wakefulness in mice^63^. The latter could be a candidate gene responsible for the reduction in sleep behavior of exposed fish 29–32 dpe, although this gene was also upregulated in infected fish. However, the few shared DEGs between exposed and infected fish could indicate that immunological and associated (neuro-) inflammatory processes differed substantially between these groups (Fig. 3B,C). Differences between exposed and infected fish have also been found in other studies, even at later stages post-exposure, such as changes in liver gene expression, body condition, and gut microbiome composition^21,39,64^, which might indicate chronic inflammation following an immune response to either the parasite or translocated bacteria in exposed fish.

Whether immune responses play a role in the observed effects of *S. solidus* infection on sleep-like behavior and the genetic and cellular pathways involved need to be further investigated. Combining analyses of the immune response at the hematopoietic site of the fish, the head kidney, with brain RNA-seq could reveal more detailed insights into the hypothesized immune-sleep interaction. Studies show that the combination of sleep-deprivation in rats with *Trichinella spiralis infections* alter several immune parameters^65,66^. Together with our findings, such studies indicate that the immune-sleep interaction could be bidirectional. Further studies should investigate whether sleep plays an essential role in fighting parasitic infections and whether manipulative parasites could reveal potentially adaptive benefits of sleep upon infection. Such interactions could also be relevant for biomedical aspects: sleep and immune-associated mediators involved in macro-parasites’ effects on sleep might even serve as candidates for novel therapeutic targets to treat macro-parasitic diseases on the one hand, and sleep disorders on the other. Furthermore, it is necessary to gain a better understanding of the sleep ecology of three-spined sticklebacks. To date, only two studies have explored rhythmicity in activity behavior during the breeding season^67^ and overall circadian rhythmicity^26^. In contrast to our findings, the latter study found stickleback to be nocturnal. However, they also observed striking individual differences in activity behavior, which is in line with our findings.

The differences in diurnal activity and sleep-like behavior among individuals might be due to the large variety of ecological conditions to which sticklebacks are adapted in nature, which has also been discussed for other animals^68^. Therefore, sleep may also be an important phenotypic mechanism for individual, temporal niche conformance under fluctuating environmental conditions^69^. Likewise, our results suggest that (macro-) parasitic infections could be an important environmental parameter that, among other things, explains the remarkable inter-individual variation but also interspecific variation in sleep throughout the animal kingdom^70^ on an ecological and evolutionary scale. Nevertheless, to our knowledge, this study is the first to specifically focus on sleep-like behavior in sticklebacks. Because of the limitations of electroencephalographic slow-wave measurements in fish, which is a common method for characterizing sleep in mammals and birds^68,71,^ our activity-based characterization of sleep with a hidden Markov model provides an objective approach for sleep research that can be easily transferred to other non-model organisms. This approach could be extended by measuring the arousal threshold of inactive individuals, which has been done in other fish species such as zebrafish and the Mexican cavefish^3,29^, to gain more certainty regarding the observed behavioral state. Therefore, investigating infection-induced changes in sleep behavior together with more detailed information about the physiology and ecology of individuals could help to better understand the role of parasites as drivers of immune-sleep interactions.

## Conclusion

Our study showed that macro-parasite infections can affect the sleep-like behavior of parasite-infected and exposed fish in differential ways and that these effects become stronger as the infection progresses. Moreover, we identified differentially expressed genes in the brains of the fish that are associated with immune responses and sleep regulation, which might be involved in the observed changes in sleep-like behaviors. These changes might have interesting ecological and evolutionary implications, which can be further explored by future studies focusing on later time points post-exposure, as well as on more detailed insights into immune response dynamics and sleep of animals to deepen our understanding of the hypothesized immune-sleep interaction.

## Material and methods

### Experimental stickleback population and husbandry

For this experiment, we used F1 offspring of wild-caught three-spined sticklebacks from Lake Constance which are commonly infected with *S. solidus*^72^. Parental fish were caught in late May 2021 using minnow traps in a river mouth and marina close to Langenargen, Germany, with permission from the Fisheries Research Station Langenargen. After 2–3 weeks of acclimatization to laboratory conditions, we collected ovaries and sperm from the wild-caught fish and artificially fertilized them. The offspring were raised in tap water at 17 °C and fed daily with freshly hatched *Artemia* larvae and frozen lobster eggs. After reaching a size of approximately 20 mm, we transferred the fish from smaller breeding tanks to 14 l aquaria with recirculating tap water, temperature control, mechanical, UV, and biological filtration (Vewa Tech, Hamm, Germany). Fish were fed frozen lobster eggs and chironomid larvae. At 4 months of age, the fish were fed only chironomid larvae. Over their entire lifespan, sticklebacks were exposed to a 15/9 h light/dark cycle with 1 h of simulated sunset and sunrise. Fish were maintained in their respective families (siblings derived from one mating pair).

### Infection with *S. solidus*

We experimentally infected sticklebacks with *S. solidus* from a stream stickleback population residing in the Ibbenbürener Aa, northwest Germany, to remove any potential effects of local adaptation. We used eggs from one breeding pair (IBB 26) for infections, which were artificially bred according to Schärer and Wedekind^73^. The eggs were incubated in Petri dishes for at least two weeks at 20 °C in the dark. We isolated male copepods from a laboratory stock of *Macrocyclops albidus* in 24-well plates two days before parasite exposure. We starved the copepods during isolation to increase the probability of parasite consumption. We induced hatching of the parasites by 3 h of light exposure in the evening, followed by 9 h of darkness and subsequent light exposure in the morning. Two to three free-swimming coracidia were collected using a pipette and transferred to each isolated copepod. Per behavioral recording of sticklebacks, we exposed 36 copepods to *S. solidus* and eight remained unexposed in the control group. After exposure, the copepods were incubated at 20 °C and fed every 48 h with 10–20 live paramecium per copepod. The copepods were maintained for at least 12 days to allow the parasite to develop into the infective procercoid stage. We then screened the copepods for procercoids under a microscope and used single-infected copepods for stickleback infection. Five families of F1 Lake Constance sticklebacks were used for the experiment. We exposed one fish per family and recorded their behavior, and the other remained unexposed for the control. At the time of exposure, the fish were between 4,5 and 6,5 months old. For each behavioral recording, we isolated 8–10 fish in jars with 400 ml block water. After 24 h of acclimatization, we exposed each 4–5 sticklebacks to an infected or non-exposed copepod in the jar (Fig. 1B). Two days before parasite exposure, the fish were starved to increase the likelihood of copepod uptake. After another 24 h, the water in the jars was filtered to determine whether the copepod was consumed by the fish. We used family pairs in which both fish ate copepods for behavioral recordings.

### Behavior recording

To measure locomotor activity and sleep-like behavior of sticklebacks, we used fully automated cameras. The principle of this system is to place fish in experimental tanks, illuminate the tanks with infrared LEDs, and monitor fish activity throughout the day and night using infrared vision cameras (Fig. 1B). We integrated four Raspberry Pi 4 cameras into an aluminum frame to horizontally cover two 3.6 l zebrafish tanks using Techniplast (Model ZB 30). These tanks have a low depth (up to 10.5 cm), which does not allow the fish to move much along a three-dimensional axis, thereby biasing two-dimensional video tracking. Moreover, the individual tanks had a vision barrier between them, so the fish were unable to see each other during the experiment. The tanks were filled with water from the same block where the fish were kept. The oxygen supply to the experimental tanks was provided by an aquarium air pump (EHEIM). Behind each tank, we vertically placed two 850 nm LED stripes with transparent paper between the tank and LEDs for background infrared illumination.

In each behavior recording session, we transferred four exposed (infected and exposed, but not infected) and four control fish individually and randomized into the tanks. All experiments started at 11:20 AM and finished 70 h 40 min later (i.e., until 10 AM on day 3 after recording started). We tested 24 exposed and 24 control fish (*n* = 48) for activity and sleep-like behavior immediately after exposure to the parasite to detect possible effects of early infection on sleep-like behavior (Fig. 1B). We euthanized and dissected half of the fish (12 exposed and 12 non-exposed) with an overdose (0.5 g/l) of tricaine methanesulfate (MS222) for further use immediately after the first sleep behavior recording. The other half of the fish (12 exposed and 12 non-exposed) were transferred back into the block and kept individually in net spawning boxes (JBL; 13.4 × 2.3 × 17.9 cm). We recorded this group of fish again within the same setting after 29 days of parasite exposure to test for possible long-term effects of infection on sleep (Fig. 1B). Thereafter, the fish were euthanized and dissected. Temperature and light conditions (17 °C, 15/9 h light/dark) did not change during parasite exposure or behavioral recording. During behavioral recordings and in the net boxes, the fish were fed once per day between 4 PM and 5 PM with frozen chironomid larvae.

### Video analysis and data processing

To track the activity and sleep-like behavior of the fish, we used the open-source Python module Phenopype^74^. Within Phenopype, we drew virtual masks defining the arena for each fish, excluding the water surface, tank walls, bottom, and left or right (depending on the tank position within the system) area of the tank where bubbles emerged from the air pumps. Therefore, these masks enabled undisturbed tracking of fish. The position of the fish was tracked five times per second. All measurements were converted from pixels to millimeters by estimating the pixel/mm ratio for each video to normalize the displacement of all fish. We then calculated the locomotor activity of each fish and estimated the displacement in mm from each frame to the next frame. After that, we calculated the sum of displacements per minute to obtain an estimate of the locomotor activity per time interval.

### Dissection and parasite screening

After recording the sleep behavior, we euthanized all fish with an overdose (0.5 g/l) of tricaine methanesulfate (MS222). We measured the total and standard lengths (from the snout to the base of the caudal fin) to the nearest millimeter and weighed the fish to the nearest milligram (Table S1). We then opened the body cavity of the fish on the ventral side with sterile scissors from the urogenital pore to the gills and screened the interior for a life tapeworm. The parasite was easy to recognize at 32 dpe, but at 4 dpe, it was still very small (approximately 100 μm^75^). Therefore, we incubated the body and organs of the fish at room temperature in saline solution (PBS) to detach the parasite from the fish tissue. We then intensively scanned the PBS-incubated organs and bodies under a binocular with a black background and bright illumination for actively moving parasites. The brains of all 48 fish were dissected, immediately frozen in liquid nitrogen, and stored at -80 °C for RNA sequencing.

### RNA extraction

Frozen brains were immediately homogenized with a cell plunger in 1 ml Ambion TRIzol reagent to avoid RNA degradation. Subsequently, the samples were sonified in an ultrasonic bath for 10 min. After centrifugation at 4 °C and 13000 rpm for 5 min, the supernatant was transferred to a new tube, and 200 ml of chloroform was added to the brain samples and incubated for 15 min at room temperature (RT). The suspension at 10500 rpm at 4 °C for 15 min, and 400 ml of the aqueous phase was transferred to a new tube to extract RNA using the Promega SV Total RNA Isolation kit, according to the corresponding protocol. Subsequently, we eluted the RNA in 80 ml nuclease-free water and stored it at -80 °C until sequencing. Four replicates were sequenced for each treatment and time point (24 samples in total). RNA quantification and qualification, library preparation, sequencing, and data analysis were performed by Biomarker Technologies (BMK) GmbH (Münster, Germany). The RNA concentration and purity were measured using a NanoDrop 2000 spectrophotometer (Thermo Fisher Scientific). RNA integrity was assessed using an RNA Nano 6000 Assay Kit on an Agilent Bioanalyzer 2100 system (Agilent Technologies, CA, USA).

### Library preparation and RNA-sequencing

A total of 1 µg of RNA per sample was used as input material for RNA sample preparation. Sequencing libraries were generated using the NEBNext UltraTM RNA Library Prep Kit for Illumina (NEB, USA), following the manufacturer’s recommendations, and index codes were added to attribute sequences to each sample. The resulting libraries were purified (AMPure XP system), and library quality was assessed using the Agilent Bioanalyzer 2100 system. Clustering of the index-coded samples was performed on a cBot Cluster Generation System using the TruSeq PE Cluster Kit v4-cBot-HS (Illumina), according to the manufacturer’s instructions. After cluster generation, the library preparations were sequenced on an Illumina Novaseq 6000 (PE150) platform and paired-end reads were generated.

### Sequencing alignment and differential expression analysis

Adaptor sequences and low-quality reads were removed from the dataset. The raw sequences were transformed into clean reads after data processing. We obtained approximately 40–58 million clean reads per sample (Supplementary Fig. 10A). Between 90.64% and 91.97% of the total reads were mapped to the NCBI reference genome sequence (GAculeatus_UGA_version5). Only reads with a perfect match or one mismatch were further analyzed and annotated based on the reference genome. More than 83% of the reads were uniquely mapped to the reference genome using Hisat2 tools, resulting in a coverage of approximately 25×per sample^76^. Differential expression analysis was performed using DESeq2. DESeq2 provides statistical routines for determining differential expression in digital gene expression data using a model based on negative binomial distribution^77^. The resulting p-values were adjusted using Benjamini and Hochberg’s approach to control the false discovery rate. Genes with an adjusted P-value < 0.05 found by DESeq2 were assigned as differentially expressed.

### GO functional enrichment analysis

Gene ontology (GO) enrichment analysis of the differentially expressed genes (DEGs) was performed using the GOseq R packages based on Wallenius non-central hyper-geometric distribution, which can adjust for gene length bias in DEGs^78^.

### Statistical analysis of sleep-like behavior

We used the observed data on locomotor activity per minute as time series data for each fish to fit a hidden Markov model (HMM) using the hmmTMB R package^79^. HMMs provide a structured and probabilistic approach for modeling sequential data with hidden underlying patterns^80^. Since sleep is strongly associated with repeated periods of behavioral inactivity^2,3^, it can be considered a hidden pattern underlying the observed sequential data of locomotor activity^81^. Therefore, HMMs enable the objective characterization of sleep-like behavior in organisms that are not suitable for procedures such as electroencephalographic analyses. For this purpose, we first assigned all missing observations of locomotor activity per minute to the NA to maintain the time-series structure. Overall, 4.33% of the observations were missing. Furthermore, we set all zero values (0.0057% of the total data points) to values slightly larger than zero, to avoid introducing an additional parameter into the model. We modeled the state-dependent distributions of locomotor activity data as gamma distributions with parameterization means and standard deviations, assuming that all individuals followed the same state-dependent process.

We used an HMM with three different observation parameters (states) to model the locomotion of each fish per min. This decision was based on comparing the overlap of state-dependent distributions of two-, three-, and four-state models and according to Pohle et al.^82^. Because of the clear differences in locomotion per minute, we interpreted state 1 as a sleep-like behavior with the lowest locomotion per minute, state 2 as moderate activity with intermediate locomotion per minute, and state 3 as high activity with the highest locomotion per minute (Fig. 2A, Supplementary Table 2). To investigate diel patterns in fish behavior, we modeled the state-switching probabilities as a function of the time of day by specifying trigonometric functions with wavelengths of 24 h as covariates and allowing different periodic effects in each condition (1–4 and 29–32 dpe of control, exposed, and infected fish, respectively). In addition, we included random intercepts per fish in each of the state-switching probabilities to account for the heterogeneity in behavior among individuals. However, we did not allow for any transitions between sleep-like behavior (state 1) and high activity (state 3) in our model formulation by fixing the respective parameters to zero. The diel activity/sleep-like patterns in each condition were investigated at the group level by inferring the periodic stationary distribution and dwell times as a function of time of day^83^ (Fig. 2C,D).

Decoding the most probable underlying state sequence for each individual using the Viterbi algorithm revealed that overall, the fish spent 18.7% in state 1, 59.1% in state 2, and 22.2% in state 3. The marginal distribution of the fitted HMM accurately captures the underlying empirical distribution (Fig. 2A). To further assess the goodness-of-fit, we simulated the data from the fitted HMM and calculated pseudo-residuals (Supplementary Fig. 4). Although the model checks revealed a slight lack of fit regarding the tails of the distribution and the observed autocorrelation, the overall model fit was satisfactory.

## Electronic supplementary material

Below is the link to the electronic supplementary material.

Supplementary Material 1

Supplementary Material 2

Supplementary Material 3

Supplementary Material 4

## Data Availability

The transcriptome sequencing data have been deposited in the National Center for Biotechnology Information’s (NCBI) Gene Expression Omnibus, https://www.ncbi.nlm.nih.gov/geo/query/acc.cgi?%20acc=GSE269460 (Geo Accession no.: GSE269460). The code for the statistical analysis is avaiable in Github (https://github.com/JaimeMAnayaRojas/Bauhus_et_al_2024.git).

## Declarations

### Statement on inclusion

Our study brings together authors from two different nationalities which are affiliated with three different universities and therefore represents an example of a fruitful collaboration across universities and nationalities.
