# Supplementary material for: Tapeworm infection affects sleep-like behavior in three-spined sticklebacks
Source: Sci Rep. 2024 Oct 8;14:23395. doi: 10.1038/s41598-024-73992-7 (PMC11461891; doi:10.1038/s41598-024-73992-7)
Supplement: Supplementary file 1 — Supplementary Material 1 [file 41598_2024_73992_MOESM1_ESM.docx]

---

title: "Results: 3-state HMM analysis"

output: html_document

date: "`r Sys.Date()`"

editor_options:

chunk_output_type: console

---

```{r setup, include=FALSE, message=FALSE}

knitr::opts_chunk$set(echo = FALSE)

options(scipen=999)

setwd("")

library(dplyr)

library(lubridate)

library(hmmTMB)

library(ggplot2)

library(knitr)

library(gridExtra)

library(ggpubr)

theme_set(theme_bw())

pal <- hmmTMB:::hmmTMB_cols

### data preparation

sticklebacks <- read.csv("regularTS1min.csv")

# create time variables

sticklebacks$time <- strptime(sticklebacks$DateTime,

format = "%Y-%m-%d %H:%M:%S")

sticklebacks$time <- as.POSIXct(sticklebacks$time)

colnames(sticklebacks)[which(colnames(sticklebacks) == "Time")] <- "Clock"

sticklebacks$Clock <- format(sticklebacks$time,

format = "%H:%M:%S")

sticklebacks$tod <- as.numeric(difftime(

as.POSIXct(sticklebacks$Clock, format = '%H:%M:%S'),

as.POSIXct('00:00', format = '%H:%M'),

units = 'min')) + 1

# change variable day

sticklebacks$Date <- as.Date(format(sticklebacks$time,

format = "%Y-%m-%d"))

df_help <- sticklebacks %>% group_by(ID) %>%

mutate(days = as.numeric(difftime(Date, Date[1],

units = 'day')) + 1,

index = 1:n()) %>% ungroup()

sticklebacks$days <- df_help$days

sticklebacks$index <- df_help$index

sticklebacks$index[which(sticklebacks$ID == "F47.1")] <-

2:(sum(sticklebacks$ID == "F47.1") + 1)

rm(df_help)

# covariates

sticklebacks$ID <- as.factor(sticklebacks$ID)

sticklebacks$Fish_id <- as.factor(sticklebacks$Fish_id)

sticklebacks$Early <- as.factor(sticklebacks$Early)

levels(sticklebacks$Early)

sticklebacks$Early <- relevel(sticklebacks$Early, ref = "1")

sticklebacks$Exposed <- as.factor(sticklebacks$Exposed)

levels(sticklebacks$Exposed)

sticklebacks$Infected <- as.factor(sticklebacks$Infected)

levels(sticklebacks$Infected)

sticklebacks <- sticklebacks %>% mutate(group = case_when(

Infected == 0 & Exposed == 0 & Early == 1 ~ 1,

Infected == 0 & Exposed == 1 & Early == 1 ~ 2,

Infected == 1 & Exposed == 1 & Early == 1 ~ 3,

Infected == 0 & Exposed == 0 & Early == 0 ~ 4,

Infected == 0 & Exposed == 1 & Early == 0 ~ 5,

Infected == 1 & Exposed == 1 & Early == 0 ~ 6

))

sticklebacks$group <- as.factor(sticklebacks$group)

sticklebacks$cosFull <- cos(2 * pi * sticklebacks$tod / 1440)

sticklebacks$sinFull <- sin(2 * pi * sticklebacks$tod / 1440)

sticklebacks$cosHalf <- cos(2 * pi * sticklebacks$tod / 720)

sticklebacks$sinHalf <- sin(2 * pi * sticklebacks$tod / 720)

# make plot of the raw data

head(sticklebacks )

getDelta <- function(Gamma, cycle = 1440, N = 3){

deltas <- matrix(NA, nrow = cycle, ncol = N)

index <- c(1:cycle,1:cycle) # index makes it easier to choose the correct Gamma in the matrix multiplications

# get Gamma_star for periodic stationary distributions

Gamma_star <- Gamma # Gamma_star will be the multiplication of the next 24 hourly(or 48 half-hourly) Gamma matrices

for (t in 1:cycle){

# loopi determines which gamma matrices will be multiplied for which t

loopi <- index[seq(t+1,cycle+t-1, by = 1)]

for (i in loopi){

Gamma_star[, , t] <- Gamma_star[, , t]%*%Gamma[,,i]

}

}

# get periodic stationary distributions

for (t in 1:cycle) {

deltas[t, ] <- solve(t(diag(N) - Gamma_star[, , t] + 1), rep(1, N))

}

return(deltas)

}

tod_mean <- function(beta, tod = 1:1440, group = 1){

beta12 <- beta[1:18]

beta13 <- beta[19:36]

beta21 <- beta[37:54]

beta23 <- beta[55:72]

beta31 <- beta[73:90]

beta32 <- beta[91:108]

group2 = ifelse(group == 2, 1, 0)

group3 = ifelse(group == 3, 1, 0)

group4 = ifelse(group == 4, 1, 0)

group5 = ifelse(group == 5, 1, 0)

group6 = ifelse(group == 6, 1, 0)

n <- length(tod)

N <- 3

covsMat <- data.frame(Intercept = 1,

group2 = group2,

group3 = group3,

group4 = group4,

group5 = group5,

group6 = group6,

cosFull = cos(2 * pi * tod / n),

sinFull = sin(2 * pi * tod / n),

int2_cosFull = cos(2 * pi * tod / n) * group2,

int3_cosFull = cos(2 * pi * tod / n) * group3,

int4_cosFull = cos(2 * pi * tod / n) * group4,

int5_cosFull = cos(2 * pi * tod / n) * group5,

int6_cosFull = cos(2 * pi * tod / n) * group6,

int2_sinFull = sin(2 * pi * tod / n) * group2,

int3_sinFull = sin(2 * pi * tod / n) * group3,

int4_sinFull = sin(2 * pi * tod / n) * group4,

int5_sinFull = sin(2 * pi * tod / n) * group5,

int6_sinFull = sin(2 * pi * tod / n) * group6)

Gamma12 <- beta12 %*% t(as.matrix(covsMat))

Gamma13 <- beta13 %*% t(as.matrix(covsMat))

Gamma21 <- beta21 %*% t(as.matrix(covsMat))

Gamma23 <- beta23 %*% t(as.matrix(covsMat))

Gamma31 <- beta31 %*% t(as.matrix(covsMat))

Gamma32 <- beta32 %*% t(as.matrix(covsMat))

Gammas <- array(1, dim = c(N, N, n))

Gammas[1, 2, ] <- exp(Gamma12)

Gammas[1, 3, ] <- exp(Gamma13)

Gammas[2, 1, ] <- exp(Gamma21)

Gammas[2, 3, ] <- exp(Gamma23)

Gammas[3, 1, ] <- exp(Gamma31)

Gammas[3, 2, ] <- exp(Gamma32)

for (i in 1:n) {

Gammas[, , i] <- Gammas[, , i] / rowSums(Gammas[, , i])

}

delta <- getDelta(Gammas)

# delta <- matrix(NA, n, N)

# for(i in 1:n){

# delta[i, ] <- solve(t(diag(N) - Gammas[, , i] + 1), rep(1, N))

# }

return(delta)

}

```

```{r}

# plot of the raw data

head(sticklebacks)

sticklebacks$Treatment <- as.factor(paste(sticklebacks$Exposed,sticklebacks$Infected))

levels(sticklebacks$Treatment) <- c("Control", "Exposed", "Infected")

sticklebacks$Stage <- as.factor(sticklebacks$Early)

levels(sticklebacks$Stage) <- c("Early", "Late")

# summarize data by ID, Treatment, and Stage

head(sticklebacks)

sticklebacks %>%

group_by(Treatment, Stage, tod) %>%

summarise(mean = mean(Loco_sum, na.rm = TRUE), sd = sd(Loco_sum)) -> df_plot

p <- ggplot(data = df_plot, aes(x = tod, y = mean, colour = Treatment, group = Treatment)) +

# geom_point(alpha = 0.5) +

geom_line(alpha = 0.5, linewidth = 0.75) +

# geom_line(aes(y = mean + sd)) +

scale_color_manual(values = c("black","#78baed","#D55E00")) +

# scale_fill_manual(values = pal[c(1,3,2)]) +

scale_x_continuous(name = "Time of day (Hours)",

breaks = seq(0, 1440, by = 120),

labels = seq(0, 24, by = 2)) +

scale_y_continuous(name = "Locomotor activity (m/min)",

limits = c(0, 4)) +

facet_grid(Stage ~ .) +

theme(legend.position = "none")

p <- p + theme_classic() + theme(legend.position = "bottom", # Move legend to bottom

legend.direction = "horizontal")

# add vertical shaded areas for night time

p + geom_rect(aes(xmin = 0, xmax = 360+30, ymin = -Inf, ymax = Inf),

fill = "gray", alpha = 0.01, color =NA) +

geom_rect(aes(xmin = 1290, xmax = 1440, ymin = -Inf, ymax = Inf),

fill = "gray", alpha = 0.01, color =NA)

ggsave("plots/locoSum_1a.pdf", width = 20, height = 9, units = "cm")

```

I used the same data preparation and model formulation as detailed in the first report.

Shortly summarised, we assume all individuals to follow the same state-dependent distributions, modelled as gamma distributions formulated in terms of a mean and standard deviation.

For the 3-state model, transitions between states 1 and 3 are prohibited by fixing the respective parameters to zero (instead of estimating those parameters), meaning that we do not allow any transitions between the least active and the most active state.

This restriction solves all problems that previously occurred with respect to model estimation (in particular, numerical instability).

All models were fitted using the recent R package hmmTMB (reference: Michelot, 2022. hmmTMB: Hidden Markov models with flexible covariate effects in R. arXiv:2211.14139).

## 3-state vs. 4-state HMM

The following plot shows the estimated state-dependent distributions of the final 3-state model (each weighted according to their proportion of decoded states):

<!-- (I do not include the transition probability matrix, as we now model the state transition probabilities as a function of covariates --- see next section). -->

```{r, warning=FALSE, fig.width=8, fig.height=4, fig.align='center', cache=TRUE}

# 3 states

hmm3N <- readRDS("3stateHMM_restrict_group&tod_int_reFishID.rds")

sticklebacks$states <- hmm3N$viterbi()

z <- seq(min(sticklebacks$Loco_sum, na.rm = TRUE), 10, by = 0.01)

weight <- table(sticklebacks$states) / nrow(sticklebacks)

mu <- hmm3N$par()$obspar[1, , 1]

sigma <- hmm3N$par()$obspar[2, , 1]

gamma1 <- weight[1] * dgamma(z, shape = mu[1]^2 / sigma[1]^2,

scale = sigma[1]^2 / mu[1])

gamma2 <- weight[2] * dgamma(z, shape = mu[2]^2 / sigma[2]^2,

scale = sigma[2]^2 / mu[2])

gamma3 <- weight[3] * dgamma(z, shape = mu[3]^2 / sigma[3]^2,

scale = sigma[3]^2 / mu[3])

marginal <- gamma1 + gamma2 + gamma3

df <- data.frame(z = z, gamma1 = gamma1, gamma2 = gamma2, gamma3 = gamma3,

marginal = marginal)

colors <- c("State 1 (sleep)" = pal[1], "State 2 (moderate activity)" = pal[3], "State 3 (high activity)" = pal[2],

"marginal" = "black")

p <- ggplot() +

geom_histogram(aes(x = Loco_sum, y = after_stat(density)), data = sticklebacks,

breaks = seq(0, 10, by = 0.25), fill = "lightgray", color = "lightgray") +

geom_line(aes(x = z, y = gamma1, color = "State 1 (sleep)"), data = df,

linewidth = 1) +

geom_line(aes(x = z, y = gamma2, color = "State 2 (moderate activity)"), data = df,

linewidth = 1) +

geom_line(aes(x = z, y = gamma3, color = "State 3 (high activity)"), data = df,

linewidth = 1) +

geom_line(aes(x = z, y = marginal, color = "marginal"), data = df,

linewidth = 1, linetype = "dotted") +

scale_color_manual(values = colors, name = "") +

scale_y_continuous(limits = c(0, 0.75))

p + theme_classic() + ylab("Density") + xlab("Locomotor activity (m/min)") +

theme(legend.position = c(0.8, 0.8))

ggsave("plots/states_1b.pdf", width = 15, height = 15, units = "cm")

hmm3N$print_obspar()

# hmm3N$print_tpm()

```

For comparison, I also include a basic 4-state HMM (i.e. without covariates) fitted to the data:

```{r, warning=FALSE, fig.width=8, fig.height=4, fig.align='center', cache=TRUE}

# 4 states

hmm4N <- readRDS("4stateHMM.rds") # "4stateHMM_finalData.rds"

sticklebacks$states4N <- hmm4N$viterbi()

weight4N <- table(sticklebacks$states4N) / nrow(sticklebacks)

mu <- hmm4N$par()$obspar[1, , 1]

sigma <- hmm4N$par()$obspar[2, , 1]

gamma1 <- weight4N[1] * dgamma(z, shape = mu[1]^2 / sigma[1]^2,

scale = sigma[1]^2 / mu[1])

gamma2 <- weight4N[2] * dgamma(z, shape = mu[2]^2 / sigma[2]^2,

scale = sigma[2]^2 / mu[2])

gamma3 <- weight4N[3] * dgamma(z, shape = mu[3]^2 / sigma[3]^2,

scale = sigma[3]^2 / mu[3])

gamma4 <- weight4N[4] * dgamma(z, shape = mu[4]^2 / sigma[4]^2,

scale = sigma[4]^2 / mu[4])

marginal <- gamma1 + gamma2 + gamma3 + gamma4

df <- data.frame(z = z, gamma1 = gamma1, gamma2 = gamma2, gamma3 = gamma3,

gamma4 = gamma4, marginal = marginal)

colors <- c("state 1" = pal[1], "state 2" = pal[2], "state 3" = pal[3],

"state 4" = pal[4], "marginal" = "black")

p <- ggplot() +

geom_histogram(aes(x = Loco_sum, y = after_stat(density)), data = sticklebacks,

breaks = seq(0, 10, by = 0.25), fill = "gray", color = "white") +

geom_line(aes(x = z, y = gamma1, color = "state 1"), data = df,

linewidth = 0.8) +

geom_line(aes(x = z, y = gamma2, color = "state 2"), data = df,

linewidth = 0.8) +

geom_line(aes(x = z, y = gamma3, color = "state 3"), data = df,

linewidth = 0.8) +

geom_line(aes(x = z, y = gamma4, color = "state 4"), data = df,

linewidth = 0.8) +

geom_line(aes(x = z, y = marginal, color = "marginal"), data = df,

linewidth = 0.8, linetype = "dashed") +

scale_color_manual(values = colors, name = "") +

scale_y_continuous(limits = c(0, 0.75)) +

theme(legend.position = c(0.8, 0.7))

p

hmm4N$print_obspar()

hmm4N$print_tpm()

```

Regarding the 4-state model, there is substantial overlap in the state-dependent distributions and, in particular, state 2 and state 3 hardly seem to differ qualitatively (as both states might relate to the same or a similar behaviour).

In the following, I will thus focus on the 3-state HMM.

## Decoded states

I decoded the most probable underlying state sequence for each individual using the Viterbi algorithm.

The overall percentage of decoded states is `r round(weight[1] * 100, digits = 1)`% in state 1, `r round(weight[2] * 100, digits = 1)`% in state 2, and `r round(weight[3] * 100, digits = 1)`% in state 3.

The following plots show the percentages of time spent in each state for all individuals, partitioned into early and late observation period (corresponding to upper and lower row, respectively) as well as experimental condition (i.e. control, exposed, infected).

```{r, fig.align='center', fig.width=10, fig.height=6, cache=TRUE}

sticklebacks$stateName <- as.factor(sticklebacks$states)

levels(sticklebacks$stateName) <- c("state 1", "state 2", "state 3")

sticklebacks <- sticklebacks %>% group_by(ID) %>%

mutate(p_state1 = sum(states == 1) / n(),

p_state2 = sum(states == 2) / n(),

p_state3 = sum(states == 3) / n())

pBase <- ggplot(mapping = aes(reorder(Fish_id, p_state1))) +

scale_fill_manual(values = pal[c(1,3,2)]) + labs(y = "percentage", x = "ID") +

theme(legend.title = element_blank())

pNot1 <- pBase + geom_bar(data = filter(sticklebacks,

Early == 1 & Infected == 0 & Exposed == 0),

aes(fill = stateName), position = "fill") +

ggtitle("early control")

pExp1 <- pBase + geom_bar(data = filter(sticklebacks,

Early == 1 & Infected == 0 & Exposed == 1),

aes(fill = stateName), position = "fill") +

ggtitle("early exposed")

pInf1 <- pBase + geom_bar(data = filter(sticklebacks,

Early == 1 & Infected == 1 & Exposed == 1),

aes(fill = stateName), position = "fill") +

ggtitle("early infected")

pNot0 <- pBase + geom_bar(data = filter(sticklebacks,

Early == 0 & Infected == 0 & Exposed == 0),

aes(fill = stateName), position = "fill") +

ggtitle("late control")

pExp0 <- pBase + geom_bar(data = filter(sticklebacks,

Early == 0 & Infected == 0 & Exposed == 1),

aes(fill = stateName), position = "fill") +

ggtitle("late exposed")

pInf0 <- pBase + geom_bar(data = filter(sticklebacks,

Early == 0 & Infected == 1 & Exposed == 1),

aes(fill = stateName), position = "fill") +

ggtitle("late infected")

ggarrange(pNot1, pExp1, pInf1, pNot0, pExp0, pInf0, nrow = 2, ncol = 3,

common.legend = TRUE, legend = "bottom")

```

The previous plot reflects the high variation in activity (levels) between the individuals, which we would like to address in our model using random effects (see later sections).

As main interest lies in the activity patterns of the fish, the following plot shows the percentage of individuals in the three states at each observation time, plotted over the whole period and separated into the different conditions:

```{r, fig.width=10, fig.height=6, fig.align='center', cache=TRUE}

# New facet label names

early.labs <- c("Early", "Late")

names(early.labs) <- c("1", "0")

exp.labs <- c("Control", "Exposed")

names(exp.labs) <- c("0", "1")

inf.labs <- c("Not infected", "Infected")

names(inf.labs) <- c("0", "1")

p2 <- ggplot(sticklebacks, aes(x = index, color = stateName, fill = stateName)) +

geom_bar(position = "fill") +

scale_color_manual(values = pal[c(1,3,2)]) +

scale_fill_manual(values = pal[c(1,3,2)] ) + #pal[c(1,3,2)]

labs(y="State proportion") +

facet_grid(Exposed + Infected ~ Early,

labeller = labeller(Early = early.labs,

Exposed = exp.labs,

Infected = inf.labs)) +

scale_x_continuous(name = "Time of the day (H:min)",

breaks = seq(0, 4000, by = 360),

labels = rep(c("15:00", "21:00", "3:00", "9:00"), 3)) +

theme(legend.position = "bottom", legend.title = element_blank(),

axis.text.x = element_text(angle = 45, hjust = 1),

panel.grid.major = element_blank(),

panel.grid.minor = element_blank())

p2

# add vertical shaded areas for the night time

# remove the stroke from the shaded area

p2 + geom_rect(aes(xmin = 390, xmax = 930, ymin = 0, ymax = 1),

fill = "grey", alpha = 0.02, color = NA) +

geom_rect(aes(xmin = 1830, xmax = 2370, ymin = 0, ymax = 1),

fill = "grey", alpha = 0.02, color = NA) +

geom_rect(aes(xmin = 3270, xmax = 3810, ymin = 0, ymax = 1),

fill = "grey", alpha = 0.02, color = NA)

```{r, fig.width=10, fig.height=6

ggsave("state1_proportions_edit3.svg", width=20, height=15, dpi=500)

ggsave("plots/states_1cnew.pdf", width = 20, height = 15, units = "cm")

```

```

From these plots, some differences between conditions can be detected as well as time periods (over a day) which appear to be more active/inactive, on average.

Regarding sleeping behaviour (i.e. state 1), differences in diel patterns between the groups can also be visualised in circular bar plots, as done in the following.

These plots show the percentage of state 1 averaged over all fish of a group and each time point, highlighting, for example, that fish in the late exposed condition generally occupy state 1 less often:

```{r, fig.width=10, fig.height=6, fig.align='center', warning=FALSE, message=FALSE, cache=TRUE}

# circular plots

props <- sticklebacks %>% group_by(group, tod) %>%

summarise(pState1 = sum(states == 1) / n())

props$PState1 <- props$pState1*100

pBase <- ggplot(mapping = aes(tod, pState1)) +

coord_polar(theta = "x") + ylab("Sleep proportion") +

scale_y_continuous(limits = c(0, 0.8)) +

scale_x_continuous(name = "Time of the day",

breaks = seq(0, 1440, by = 60),

labels = c(0:23, 0))

p1 <- pBase + geom_bar(data = filter(props, group == 1),

stat = "identity", col = pal[1]) +

ggtitle("Early control") +

geom_point(aes(x = 0, y = 0), col = "white") # , shape = 4, stroke = 2

p2 <- pBase + geom_bar(data = filter(props, group == 2),

stat = "identity", col = pal[1]) +

ggtitle("Early exposed") +

geom_point(aes(x = 0, y = 0), col = "white")

p3 <- pBase + geom_bar(data = filter(props, group == 3),

stat = "identity", col = pal[1]) +

ggtitle("Early infected") +

geom_point(aes(x = 0, y = 0), col = "white")

p4 <- pBase + geom_bar(data = filter(props, group == 4),

stat = "identity", col = pal[1]) +

ggtitle("Late control") +

geom_point(aes(x = 0, y = 0), col = "white")

p5 <- pBase + geom_bar(data = filter(props, group == 5),

stat = "identity", col = pal[1]) +

ggtitle("Late exposed") +

geom_point(aes(x = 0, y = 0), col = "white")

p6 <- pBase + geom_bar(data = filter(props, group == 6),

stat = "identity", col = pal[1]) +

ggtitle("Late infected") +

geom_point(aes(x = 0, y = 0), col = "white")

ggarrange(p1, p2, p3, p4, p5, p6, ncol = 3, nrow = 2)

ggsave("plots/states_1d.pdf", width = 20, height = 15, units = "cm")

```

```

```

The following section investigates these differences in activity levels between the conditions as well as diel patterns in more detail, based on the estimated covariate effects.

## Covariates

To analyse diel variation in the state-switching dynamics, I model the state transition probabilities as a function of time of day using trigonometric functions, allowing for different periodic effects in each condition.

The following plots display the probability (i.e. expected percentage) of occupying either of the three states over the time of day (left: state 1, middle: state 2, right: state 3), as implied by the periodic stationarity of the fitted HMM, and additionally include 95% point-wise confidence intervals (CIs) to visualise uncertainty in the estimated effects.

<!-- as implied by the time-varying transition probability matrix (t.p.m.) -->

The upper row corresponds to the early period, the lower row to the late one.

(Please let me know if you have suggestions for different colors.)

```{r, fig.width=10, fig.height=6, fig.align='center', cache=TRUE}

cbPalette <- c("black", "#E69F00", "#56B4E9", "#009E73", "#F0E442",

"#0072B2", "#D55E00", "#CC79A7")

newdata <- read.csv("stateProbs_int_group&tod_reFishID.csv")

newdata$Early <- as.factor(ifelse(newdata$group == 1 | newdata$group == 2 |

newdata$group == 3, 1, 0))

newdata$Early <- relevel(newdata$Early, ref = "1")

newdata$group <- as.factor(newdata$group)

pBase <- ggplot(mapping = aes(x = tod, group = group,

color = group, fill = group)) +

scale_x_continuous(name = "time of day (hours)",

breaks = seq(0, 1440, by = 120),

labels = seq(0, 24, by = 2)) +

scale_y_continuous(limits = c(0, 1),

breaks = seq(0, 1, by = 0.2)) +

scale_color_manual(name = "condition", values = cbPalette[c(1, 6, 7)],

labels = c("control", "exposed", "infected")) +

scale_fill_manual(name = "condition", values = cbPalette[c(1, 6, 7)],

labels = c("control", "exposed", "infected"))

pDelta1_1 <- pBase + geom_line(aes(y = state1), linewidth = 0.8,

data = filter(newdata, Early == 1)) +

geom_ribbon(aes(ymin = lci1, ymax = uci1), alpha = 0.2,

data = filter(newdata, Early == 1)) +

ylab("probability of state 1") + ggtitle("Early")

pDelta2_1 <- pBase + geom_line(aes(y = state2), linewidth = 0.8,

data = filter(newdata, Early == 1)) +

geom_ribbon(aes(ymin = lci2, ymax = uci2), alpha = 0.2,

data = filter(newdata, Early == 1)) +

ylab("probability of state 2") + ggtitle("Early")

pDelta3_1 <- pBase + geom_line(aes(y = state3), linewidth = 0.8,

data = filter(newdata, Early == 1)) +

geom_ribbon(aes(ymin = lci3, ymax = uci3), alpha = 0.2,

data = filter(newdata, Early == 1)) +

ylab("probability of state 3") + ggtitle("Early")

pDelta1_0 <- pBase + geom_line(aes(y = state1), linewidth = 0.8,

data = filter(newdata, Early == 0)) +

geom_ribbon(aes(ymin = lci1, ymax = uci1), alpha = 0.2,

data = filter(newdata, Early == 0)) +

ylab("probability of state 1") + ggtitle("Late")

pDelta2_0 <- pBase + geom_line(aes(y = state2), linewidth = 0.8,

data = filter(newdata, Early == 0)) +

geom_ribbon(aes(ymin = lci2, ymax = uci2), alpha = 0.2,

data = filter(newdata, Early == 0)) +

ylab("probability of state 2") + ggtitle("Late")

pDelta3_0 <- pBase + geom_line(aes(y = state3), linewidth = 0.8,

data = filter(newdata, Early == 0)) +

geom_ribbon(aes(ymin = lci3, ymax = uci3), alpha = 0.2,

data = filter(newdata, Early == 0)) +

ylab("probability of state 3") + ggtitle("Late")

ggarrange(pDelta1_1, pDelta2_1, pDelta3_1,

pDelta1_0, pDelta2_0, pDelta3_0, nrow = 2, ncol = 3,

common.legend = TRUE, legend="bottom")

```

To facilitate comparison of the effects between early and late period for each condition, the following plots show the same results as the previous ones but with one plot for each condition (instead of for each period):

```{r, fig.width=10, fig.height=8, fig.align='center', cache=TRUE}

pBase <- ggplot(mapping = aes(x = tod, group = Early,

color = Early, fill = Early)) +

scale_x_continuous(name = "time of day (hours)",

breaks = seq(0, 1440, by = 120),

labels = seq(0, 24, by = 2)) +

scale_y_continuous(limits = c(0, 1),

breaks = seq(0, 1, by = 0.2)) +

scale_color_manual(name = "period", values = cbPalette[2:3],

labels = c("early", "late")) +

scale_fill_manual(name = "period", values = cbPalette[2:3],

labels = c("early", "late"))

pDelta1_1 <- pBase + geom_line(aes(y = state1), linewidth = 0.8,

data = filter(newdata, group == 1 | group == 4)) +

geom_ribbon(aes(ymin = lci1, ymax = uci1), alpha = 0.2,

data = filter(newdata, group == 1 | group == 4)) +

ylab("probability of state 1") + ggtitle("control")

pDelta2_1 <- pBase + geom_line(aes(y = state2), linewidth = 0.8,

data = filter(newdata, group == 1 | group == 4)) +

geom_ribbon(aes(ymin = lci2, ymax = uci2), alpha = 0.2,

data = filter(newdata, group == 1 | group == 4)) +

ylab("probability of state 2") + ggtitle("control")

pDelta3_1 <- pBase + geom_line(aes(y = state3), linewidth = 0.8,

data = filter(newdata, group == 1 | group == 4)) +

geom_ribbon(aes(ymin = lci3, ymax = uci3), alpha = 0.2,

data = filter(newdata, group == 1 | group == 4)) +

ylab("probability of state 3") + ggtitle("control")

pDelta1_2 <- pBase + geom_line(aes(y = state1), linewidth = 0.8,

data = filter(newdata, group == 2 | group == 5)) +

geom_ribbon(aes(ymin = lci1, ymax = uci1), alpha = 0.2,

data = filter(newdata, group == 2 | group == 5)) +

ylab("probability of state 1") + ggtitle("exposed")

pDelta2_2 <- pBase + geom_line(aes(y = state2), linewidth = 0.8,

data = filter(newdata, group == 2 | group == 5)) +

geom_ribbon(aes(ymin = lci2, ymax = uci2), alpha = 0.2,

data = filter(newdata, group == 2 | group == 5)) +

ylab("probability of state 2") + ggtitle("exposed")

pDelta3_2 <- pBase + geom_line(aes(y = state3), linewidth = 0.8,

data = filter(newdata, group == 2 | group == 5)) +

geom_ribbon(aes(ymin = lci3, ymax = uci3), alpha = 0.2,

data = filter(newdata, group == 2 | group == 5)) +

ylab("probability of state 3") + ggtitle("exposed")

pDelta1_3 <- pBase + geom_line(aes(y = state1), linewidth = 0.8,

data = filter(newdata, group == 3 | group == 6)) +

geom_ribbon(aes(ymin = lci1, ymax = uci1), alpha = 0.2,

data = filter(newdata, group == 3 | group == 6)) +

ylab("probability of state 1") + ggtitle("infected")

pDelta2_3 <- pBase + geom_line(aes(y = state2), linewidth = 0.8,

data = filter(newdata, group == 3 | group == 6)) +

geom_ribbon(aes(ymin = lci2, ymax = uci2), alpha = 0.2,

data = filter(newdata, group == 3 | group == 6)) +

ylab("probability of state 2") + ggtitle("infected")

pDelta3_3 <- pBase + geom_line(aes(y = state3), linewidth = 0.8,

data = filter(newdata, group == 3 | group == 6)) +

geom_ribbon(aes(ymin = lci3, ymax = uci3), alpha = 0.2,

data = filter(newdata, group == 3 | group == 6)) +

ylab("probability of state 3") + ggtitle("infected")

ggarrange(pDelta1_1, pDelta1_2, pDelta1_3,

pDelta2_1, pDelta2_2, pDelta2_3,

pDelta3_1, pDelta3_2, pDelta3_3,

nrow = 3, ncol = 3,

common.legend = TRUE, legend="bottom")

```

## Expected dwell times within the states

The following plots visualise the _expected_ time spent in a specific state before switching to another one (in minutes), with early (late) period shown in the upper (lower) row and state 1 to 3 shown in each column, respectively.

```{r, fig.width=10, fig.height=6, fig.align='center', cache=TRUE}

newdata <- read.csv("expDwellTimes_CIs.csv")

newdata$group <- as.factor(newdata$group)

newdata$Late <- as.factor(ifelse(newdata$group == 1 | newdata$group == 2 |

newdata$group == 3, 0, 1))

pBase <- ggplot(mapping = aes(x = tod, group = group,

color = group, fill = group)) +

scale_x_continuous(name = "time of day (hours)",

breaks = seq(0, 1440, by = 120),

labels = seq(0, 24, by = 2)) +

scale_y_continuous(limits = c(0, 100), # 50

breaks = seq(0, 100, by = 10)) + # seq(0, 50, by = 10)

scale_color_manual(name = "condition", values = cbPalette[c(1, 6, 7)],

labels = c("control", "exposed", "infected")) +

scale_fill_manual(name = "condition", values = cbPalette[c(1, 6, 7)],

labels = c("control", "exposed", "infected"))

pDwell1_Early <- pBase + geom_line(data = filter(newdata, Late == 0),

mapping = aes(y = dstate1), linewidth = 0.8) +

geom_ribbon(aes(ymin = lci1, ymax = uci1), alpha = 0.2,

data = filter(newdata, Late == 0)) +

ylab("dwell time in state 1") + ggtitle("Early")

pDwell1_Late <- pBase + geom_line(data = filter(newdata, Late == 1),

mapping = aes(y = dstate1), linewidth = 0.8) +

geom_ribbon(aes(ymin = lci1, ymax = uci1), alpha = 0.2,

data = filter(newdata, Late == 1)) +

ylab("dwell time in state 1") + ggtitle("Late")

pDwell2_Early <- pBase + geom_line(data = filter(newdata, Late == 0),

mapping = aes(y = dstate2), linewidth = 0.8) +

geom_ribbon(aes(ymin = lci2, ymax = uci2), alpha = 0.2,

data = filter(newdata, Late == 0)) +

ylab("dwell time in state 2") + ggtitle("Early")

pDwell2_Late <- pBase + geom_line(data = filter(newdata, Late == 1),

mapping = aes(y = dstate2), linewidth = 0.8) +

geom_ribbon(aes(ymin = lci2, ymax = uci2), alpha = 0.2,

data = filter(newdata, Late == 1)) +

ylab("dwell time in state 2") + ggtitle("Late")

pDwell3_Early <- pBase + geom_line(data = filter(newdata, Late == 0),

mapping = aes(y = dstate3), linewidth = 0.8) +

geom_ribbon(aes(ymin = lci3, ymax = uci3), alpha = 0.2,

data = filter(newdata, Late == 0)) +

ylab("dwell time in state 3") + ggtitle("Early")

pDwell3_Late <- pBase + geom_line(data = filter(newdata, Late == 1),

mapping = aes(y = dstate3), linewidth = 0.8) +

geom_ribbon(aes(ymin = lci3, ymax = uci3), alpha = 0.2,

data = filter(newdata, Late == 1)) +

ylab("dwell time in state 3") + ggtitle("Late")

ggarrange(pDwell1_Early, pDwell2_Early, pDwell3_Early,

pDwell1_Late, pDwell2_Late, pDwell3_Late, nrow = 2, ncol = 3,

common.legend = TRUE, legend="bottom")

```

It appears that especially the infected fish from the late period tend to remain longer in a state during the day before switching to another one.

However, there is again quite some uncertainty in the expected dwell times and only for the most active state (i.e. state 3) in the late period, the 95% CIs from the infected fish and those from the other conditions do not overlap.

To facilitate comparison between early and late period, instead of between the conditions, the following plots show the same results but with state 1 to 3 in each row and the different conditions in each column, respectively.

```{r, fig.width=10, fig.height=8, fig.align='center', cache=TRUE}

pBase <- ggplot(mapping = aes(x = tod, group = Late,

color = Late, fill = group)) +

scale_x_continuous(name = "time of day (hours)",

breaks = seq(0, 1440, by = 120),

labels = seq(0, 24, by = 2)) +

scale_y_continuous(limits = c(0, 100),

breaks = seq(0, 100, by = 10)) +

scale_color_manual(name = "period", values = cbPalette[2:3],

labels = c("early", "late")) +

scale_fill_manual(name = "period", values = cbPalette[2:3],

labels = c("early", "late"))

pDwell1_con <- pBase + geom_line(aes(y = dstate1), linewidth = 0.8,

data = filter(newdata, group == 1 | group == 4)) +

geom_ribbon(aes(ymin = lci1, ymax = uci1), alpha = 0.2,

data = filter(newdata, group == 1 | group == 4)) +

ylab("dwell time in state 1") + ggtitle("control")

pDwell1_exp <- pBase + geom_line(aes(y = dstate1), linewidth = 0.8,

data = filter(newdata, group == 2 | group == 5)) +

geom_ribbon(aes(ymin = lci1, ymax = uci1), alpha = 0.2,

data = filter(newdata, group == 2 | group == 5)) +

ylab("dwell time in state 1") + ggtitle("exposed")

pDwell1_inf <- pBase + geom_line(aes(y = dstate1), linewidth = 0.8,

data = filter(newdata, group == 3 | group == 6)) +

geom_ribbon(aes(ymin = lci1, ymax = uci1), alpha = 0.2,

data = filter(newdata, group == 3 | group == 6)) +

ylab("dwell time in state 1") + ggtitle("infected")

pDwell2_con <- pBase + geom_line(aes(y = dstate2), linewidth = 0.8,

data = filter(newdata, group == 1 | group == 4)) +

geom_ribbon(aes(ymin = lci2, ymax = uci2), alpha = 0.2,

data = filter(newdata, group == 1 | group == 4)) +

ylab("dwell time in state 2") + ggtitle("control")

pDwell2_exp <- pBase + geom_line(aes(y = dstate2), linewidth = 0.8,

data = filter(newdata, group == 2 | group == 5)) +

geom_ribbon(aes(ymin = lci2, ymax = uci2), alpha = 0.2,

data = filter(newdata, group == 2 | group == 5)) +

ylab("dwell time in state 2") + ggtitle("exposed")

pDwell2_inf <- pBase + geom_line(aes(y = dstate2), linewidth = 0.8,

data = filter(newdata, group == 3 | group == 6)) +

geom_ribbon(aes(ymin = lci2, ymax = uci2), alpha = 0.2,

data = filter(newdata, group == 3 | group == 6)) +

ylab("dwell time in state 2") + ggtitle("infected")

pDwell3_con <- pBase + geom_line(aes(y = dstate3), linewidth = 0.8,

data = filter(newdata, group == 1 | group == 4)) +

geom_ribbon(aes(ymin = lci3, ymax = uci3), alpha = 0.2,

data = filter(newdata, group == 1 | group == 4)) +

ylab("dwell time in state 3") + ggtitle("control")

pDwell3_exp <- pBase + geom_line(aes(y = dstate3), linewidth = 0.8,

data = filter(newdata, group == 2 | group == 5)) +

geom_ribbon(aes(ymin = lci3, ymax = uci3), alpha = 0.2,

data = filter(newdata, group == 2 | group == 5)) +

ylab("dwell time in state 3") + ggtitle("exposed")

pDwell3_inf <- pBase + geom_line(aes(y = dstate3), linewidth = 0.8,

data = filter(newdata, group == 3 | group == 6)) +

geom_ribbon(aes(ymin = lci3, ymax = uci3), alpha = 0.2,

data = filter(newdata, group == 3 | group == 6)) +

ylab("dwell time in state 3") + ggtitle("infected")

ggarrange(pDwell1_con, pDwell1_exp, pDwell1_inf,

pDwell2_con, pDwell2_exp, pDwell2_inf,

pDwell3_con, pDwell3_exp, pDwell3_inf, nrow = 3, ncol = 3,

common.legend = TRUE, legend="bottom")

```

## Random effects

To account for heterogeneity in activity levels, I included random intercepts per Fish_id in the state process.

More specifically, a random intercept is included in each of the 6 state switching probabilities (i.e. the off-diagonal entries of the t.p.m.).

This allows for different state dynamics between the fish, meaning that some individuals have generally higher (or lower) probabilities of switching from one state to the other compared to the population mean.

The following plots show the probability of state 1 (reflecting sleeping behaviour) over the time of day as implied by the time-varying t.p.m. --- i.e. the same as the left column in the previous plot --- for all 6 experimental conditions, where each coloured line corresponds to an individual and the black thick line represents the population mean:

```{r, fig.width=10, fig.height=6, fig.align='center', cache=TRUE}

# random effects for Fish_id

newdata <- read.csv("stateProbs_final_reID.csv")

newdata$Fish_id <- as.factor(newdata$Fish_id)

beta <- as.vector(hmm3N$coeff_fe()$hid)

df <- data.frame(tod = 1:1440,

state1_g1 = NA, state2_g1 = NA, state3_g1 = NA,

state1_g2 = NA, state2_g2 = NA, state3_g2 = NA,

state1_g3 = NA, state2_g3 = NA, state3_g3 = NA,

state1_g4 = NA, state2_g4 = NA, state3_g4 = NA,

state1_g5 = NA, state2_g5 = NA, state3_g5 = NA,

state1_g6 = NA, state2_g6 = NA, state3_g6 = NA)

df[, 2:4] <- tod_mean(beta)

df[, 5:7] <- tod_mean(beta, group = 2)

df[, 8:10] <- tod_mean(beta, group = 3)

df[, 11:13] <- tod_mean(beta, group = 4)

df[, 14:16] <- tod_mean(beta, group = 5)

df[, 17:19] <- tod_mean(beta, group = 6)

p <- ggplot(mapping = aes(x = tod)) +

scale_x_continuous(name = "time of day (hours)",

breaks = seq(0, 1440, by = 120),

labels = seq(0, 24, by = 2)) +

scale_y_continuous(name = "probability of state 1",

limits = c(0, 1),

breaks = seq(0, 1, by = 0.2)) +

theme(legend.position="none")

p1 <- p + ggtitle("early control") +

geom_line(data = filter(newdata, group == 1),

mapping = aes(y = state1, color = Fish_id, group = Fish_id)) +

geom_line(data = df, mapping = aes(y = state1_g1), color = "black",

linewidth = 1)

p2 <- p + ggtitle("early exposed") +

geom_line(data = filter(newdata, group == 2),

mapping = aes(y = state1, color = Fish_id, group = Fish_id)) +

geom_line(data = df, mapping = aes(y = state1_g2), color = "black",

linewidth = 1)

p3 <- p + ggtitle("early infected") +

geom_line(data = filter(newdata, group == 3),

mapping = aes(y = state1, color = Fish_id, group = Fish_id)) +

geom_line(data = df, mapping = aes(y = state1_g3), color = "black",

linewidth = 1)

p4 <- p + ggtitle("late control") +

geom_line(data = filter(newdata, group == 4),

mapping = aes(y = state1, color = Fish_id, group = Fish_id)) +

geom_line(data = df, mapping = aes(y = state1_g4), color = "black",

linewidth = 1)

p5 <- p + ggtitle("late exposed") +

geom_line(data = filter(newdata, group == 5),

mapping = aes(y = state1, color = Fish_id, group = Fish_id)) +

geom_line(data = df, mapping = aes(y = state1_g5), color = "black",

linewidth = 1)

p6 <- p + ggtitle("late infected") +

geom_line(data = filter(newdata, group == 6),

mapping = aes(y = state1, color = Fish_id, group = Fish_id)) +

geom_line(data = df, mapping = aes(y = state1_g6), color = "black",

linewidth = 1)

# grid.arrange(p1, p4, p2, p5, p3, p6, nrow = 3)

grid.arrange(p1, p2, p3, p4, p5, p6, nrow = 2)

```

As can be seen, there are huge differences between individuals in their diel sleeping pattern:

some individuals have generally a much higher (or lower) probability of being in state 1 than others, resulting in high variation even within conditions.

There are also a few fish for which their sleeping behaviour hardly seems to be affected by time of day (e.g. in the late control group).

<!-- Interestingly, the sleeping pattern appears to be reversed for some individuals, with the highest probability of state 1 being active during the day (instead of nighttime), for example in the early infected and late exposed group.

To investigate this in a bit more detail, the following plots compare the decoded time series of two fish from the early infected group.

Indeed, fish 47 appears to be more active during the night than fish 21, which shows the predominant pattern in the population of being less active during nighttime. -->

The following plots compare the decoded time series of two fish from the early infected group, highlighting that even within a group, the activity patterns over time can differ greatly.

```{r, fig.width=10, fig.height=6, fig.align='center', warning=FALSE, cache=TRUE}

pBase <- ggplot(mapping = aes(x = index, xend = index, y = 0, yend = Loco_sum,

col = stateName)) +

scale_color_manual(values = pal[1:3]) +

labs(y = "Loco_sum") + # , title = "ID: F47.1"

scale_x_continuous(name = "time",

breaks = seq(0, 4000, by = 360),

labels = rep(c("15:00", "21:00", "3:00", "9:00"), 3)) +

theme(legend.position = "bottom", legend.title = element_blank())

p47 <- pBase + geom_segment(data = filter(sticklebacks, ID == "F47.1")) +

ggtitle("ID: F47.1")

p21 <- pBase + geom_segment(data = filter(sticklebacks, ID == "F21.1")) +

ggtitle("ID: F21.1")

ggarrange(p47, p21, nrow = 2, ncol = 1,

common.legend = TRUE, legend="bottom")

# # plots for representative fish per group

# p1 <- pBase + geom_segment(data = filter(sticklebacks, ID == "F38.1"))

# p2 <- pBase + geom_segment(data = filter(sticklebacks, ID == "F29.1")) # F7.1

# p3 <- pBase + geom_segment(data = filter(sticklebacks, ID == "F19.1"))

# p4 <- pBase + geom_segment(data = filter(sticklebacks, ID == "F2.0")) # F20.0

# p5 <- pBase + geom_segment(data = filter(sticklebacks, ID == "F13.0")) # F5.0

# p6 <- pBase + geom_segment(data = filter(sticklebacks, ID == "F21.0")) # F4.0

#

# ggarrange(p1, p2, p3, p4, p5, p6, nrow = 3, ncol = 2,

# common.legend = TRUE, legend="bottom")

```

## Model checking

To assess the goodness-of-fit, we can simulate data from the fitted model and check whether these synthetic observations replicate the patterns found in the real data. In the following plots, I show histograms of the minimum, maximum, median, and quartiles of the simulated observations as well as the correlation between consecutive observations based on 100 simulated data sets. The vertical line represents the value observed in the real data (the one for autocorrelation is `r round(cor(sticklebacks$Loco_sum[-1], sticklebacks$Loco_sum[-nrow(sticklebacks)], use = "complete.obs"), digits = 3)`, but not shown in the plot).

```{r, fig.width=10, fig.height=6, fig.align='center', warning=FALSE, cache=TRUE}

checks <- readRDS("modelChecks.rds")

checks$plot

```

When considering the scales of the x axis, we can conclude that the fitted model reproduces the general distribution of the observations well, with the summary statistics of the simulated data sets being slightly lower than the ones from the real data.

Only the autocorrelation of the observation process is clearly lower than observed in the real data, meaning that the fitted model does not adequately capture the correlation between consecutive observations.

A more comprehensive formal check of the model is to calculate the so-called pseudo-residuals under the fitted HMM, which are standard normally distributed if the model is correct.

The following plots assess the normality of the pseudo-residuals, where the red line corresponds to the standard normal distribution:

```{r, fig.width=12, fig.height=6, fig.align='center', cache=TRUE}

par(mfrow = c(1, 2))

pr <- readRDS("pseudoResiduals.rds")

qqnorm(pr, ylim = c(-4, 4))

abline(0, 1, col = 2, lwd = 2)

hist(pr, breaks = 40, prob = TRUE, main = "Histogram of pseudo-residuals",

ylab = "density", xlab = "pseudo-residuals")

curve(dnorm(x), col = 2, lwd = 2, add = TRUE)

```

The plots indicate a lack of fit with regard to the tails of the state-dependent distributions.

As already suggested by the marginal distribution of the fitted model (cf. first plot in this report) as well as the simulated data, we would expect more small (and large) observations under the estimated model than are observed in the data.

One explanation for this is that we have a few large observations in the data (i.e. larger than 10) and to allow for such large observations, the estimated distribution has a fat right tail, even though there are only very few large observations.

However, it is generally difficult to adequately capture the tails of a distribution, meaning that the overall model fit can still be deemed satisfactory.
