# Supplementary material for: Tapeworm infection affects sleep-like behavior in three-spined sticklebacks
Source: Sci Rep. 2024 Oct 8;14:23395. doi: 10.1038/s41598-024-73992-7 (PMC11461891; doi:10.1038/s41598-024-73992-7)
Supplement: Supplementary file 2 — Supplementary Material 2 [file 41598_2024_73992_MOESM2_ESM.docx]

**Supplemental information**

**Supplementary tables:**

**Supplementary Table 1:** Fish data with information about parasite exposure, sex, total and standard length, weight, infection status, and parasite mass. Total length was measured from the snout to the end of the caudal fin and standard length (in brackets) was measured from the snout to the caudal peduncle. Missing values are indicated with NA.

**Supplementary table 2:** Mean and standard deviation (SD) of the observation parameters (states) estimated by the HMM.

|  | State 1 | State 2 | State 3 |
| --- | --- | --- | --- |
| Mean locomotor activity (m/min) | 0.304 | 0.987 | 2.703 |
| SD locomotor activity (m/min) | 0.297 | 0.773 | 1.954 |

**Supplementary table 3:** Immune- and sleep-associated genes are differentially expressed in exposed and infected fish. Table containing the NCBI transcript ID, annotation and short summary of (putative) biological function

| **Transcript ID** | **Annotation** | **Function** |  |
| --- | --- | --- | --- |
|  |  |  |  |
|  |  |  |  |
| ccl20b | chemokine (C-C motif) ligand 20b | aggravates neuroinflammation, chemokine binding, cytokine response, leukocyte chemotaxis [1,2] |  |
| LOC120833362 | interferon-induced protein 44-like | macrophage differentiation, cytokine secretion [3] |  |
| cxcl12a | chemokine (C-X-C motif) ligand  12a (stromal cell-derived factor 1) | CNS development, immune cell regulation,  maintenance of CNS physiological function [4] |  |
| LOC120819735 | 5-hydroxytryptamine receptor 3A-like | serotonin receptor, fast depolarization of neurons, REMS regulation [5,6] |  |
| LOC120830364 | C-C motif chemokine 19-like | pro-inflammatory state, leukocyte recruitment,  CCR7 receptor binding [7] |  |
| tbxa2r | thromboxane A2 receptor | regulation of inflammatory response, regulation of  microglia, sleep associated [8,9] |  |
| LOC120814037 | protein NLRC3-like | PAMP associated,regulation of inflammatory signaling, microglia homeostasis [10] |  |
| LOC120832361 | gastrin-releasing peptide | neuropeptide activity, SCN associated [11,12] |  |
| csf1ra | colony stimulating factor 1 receptor, a | neuroinflammation, macrophage chemotaxis &  differentiation, sleep associated [13,14] |  |
| irak4 | interleukin-1 receptor-associated kinase 4 | innate immune response, IL-1 and TLR- signaling,  optimal IL-1 signal transduction [15] |  |
| LOC120815006 | interleukin-17C-like | inflammatory cytokine, downstream signal of TLR5 and  MyD88 pathogen recognition [16] |  |
| LOC120811957 | histone-lysine N-methyltransferase EZH2 | circadian clock regulation, hematopoiesis [17] |  |
| LOC120815269 | 15-hydroxyprostaglandin  dehydrogenase [NAD(+)]-like | inactivation of cellular prostaglandins [18] |  |
| LOC120827665 | interferon-induced very large GTPase 1-like | host resistance to pathogens [19] |  |
| LOC120823024 | prostaglandin reductase 1-like | inactivation of prostaglandins [20] |  |
| LOC120816500 | cerebellin-4-like | formation and maintenance of inhibitory  GABAergic connections [21] |  |
| LOC120827101 | glutathione S-transferase A-like | inactivation of exogenous and endogenous  compounds such as prostaglandins [22] |  |
| LOC120809062 | mucosa-associated lymphoid tissue  lymphoma translocation protein 1-like | promotes inflammation by recruitment of  inflammatory cytokines, T-cell activation [23] |  |
| npffl | neuropeptide FF-amide peptide precursor like | neuropeptide activity, involved in  neuroinflammatory processes [24,25] |  |
| ly97.3 | lymphocyte antigen 97, tandem duplicate 3 | LPS binding, defense response to bacteria [26] |  |
| LOC120809389 | agouti related neuropeptide | induction of feeding behavior, suppression of sleep  by hunger, promotes wakefulness [27] |  |
| LOC120815181 | protein NLRC3-like | PAMP associated, regulation of inflammatory  signaling, microglia homeostasis [10] |  |
| nmbb | neuromedin Bb | facilitates neuropeptide activity, hormone secretion,  GRP associated [12,28] |  |
| fabp4b | fatty acid binding protein 4b | upstream or within response to prostaglandin [29] |  |
| LOC120812655 | nuclear receptor ROR-alpha A-like | regulation of circadian clock, sleep duration,  anti-inflammatory, T-cell differentiation [30] |  |
| LOC120808512 | toll-like receptor 5 | PRR, recognition of bacterial flagellin,  induction of proinflammatory response [31] |  |
| LOC120819278 | peroxiredoxin-like 2A | negative regulation of macrophage induced inflammation [32] |  |
| LOC120812245 | zinc finger homeobox protein 3-like | maintenance of circadian rythm, sleep modulation [33] |  |
| LOC120835291 | adenosine receptor A1-like | sleep promotion, sleep homeostasis, inhibition of  wake-promoting neurons [34] |  |
| m17 | IL-6 subfamily cytokine M17 | macrophage proliferation and differentiation,  inflammation, sleep promoting [35,36] |  |
| LOC120812675 | class E basic helix-loop-helix protein 41-like | control of circadian rythm, short sleep  phenotype associated [37,38] |  |

**Supplementary Figures**

**
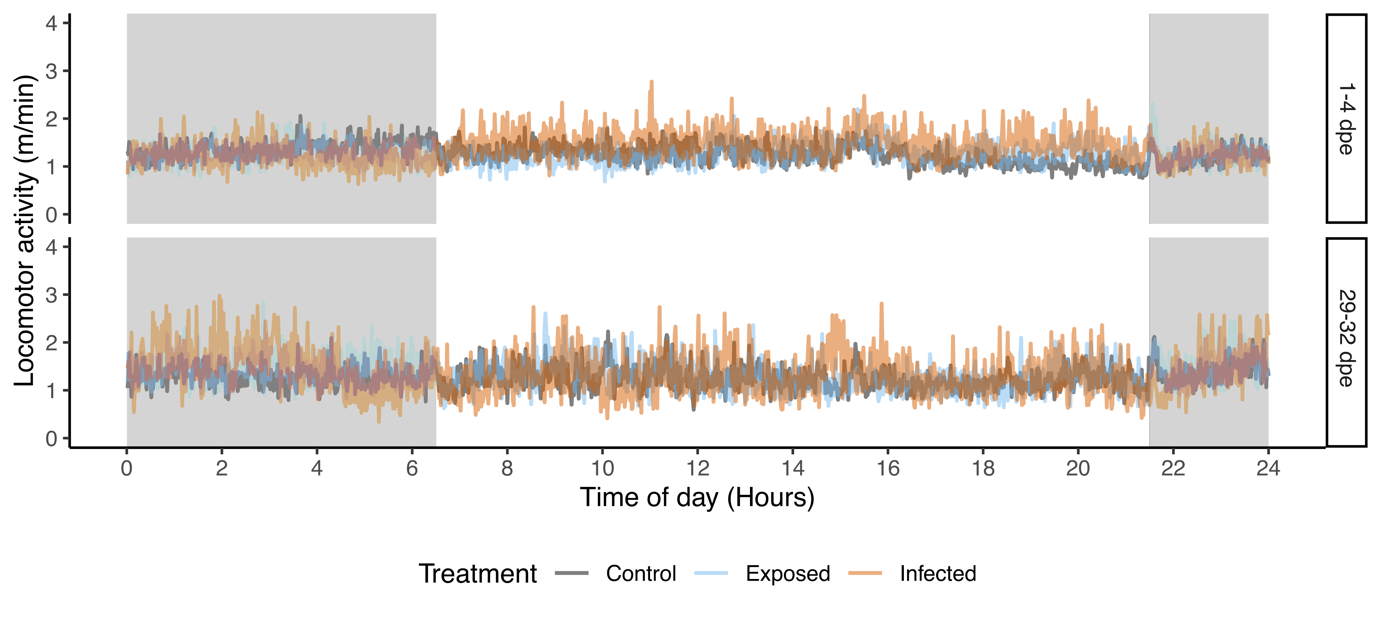
**

**Supplementary Figure 1:** raw locomotor activity data plotted as the mean locomotor activity (m/min) of the control (black), exposed (blue), and infected (red) fish averaged over 24 h for 1-4 and 29-32 days post parasite exposure (dpe). Shaded areas indicate nighttime.


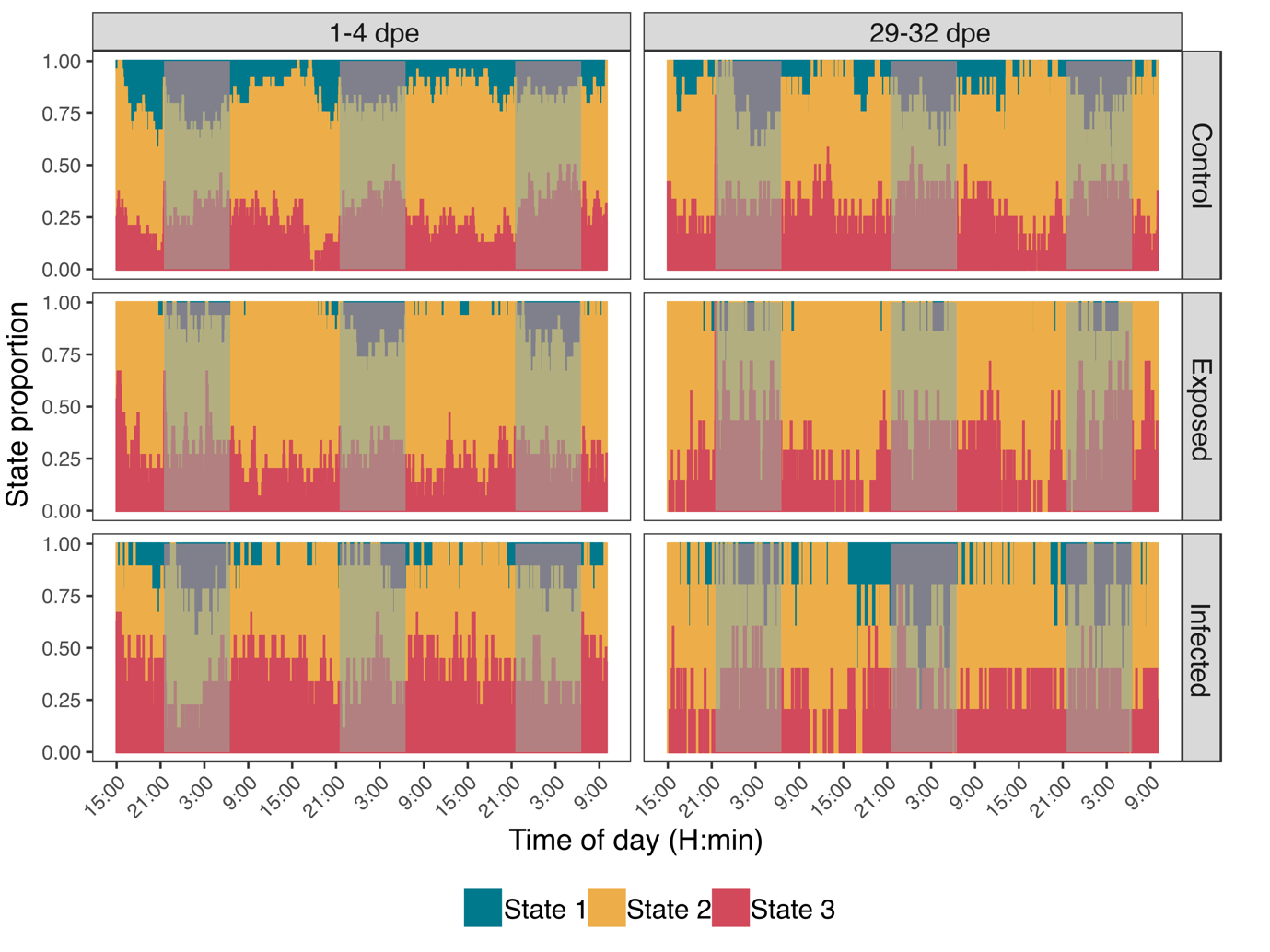


**Supplementary Figure 2:** Mean HMM state proportions of control, exposed, and infected fish over the complete recording time span 1-4 and 29-32 days post parasite exposure (dpe). Nighttime is indicated by shaded areas.


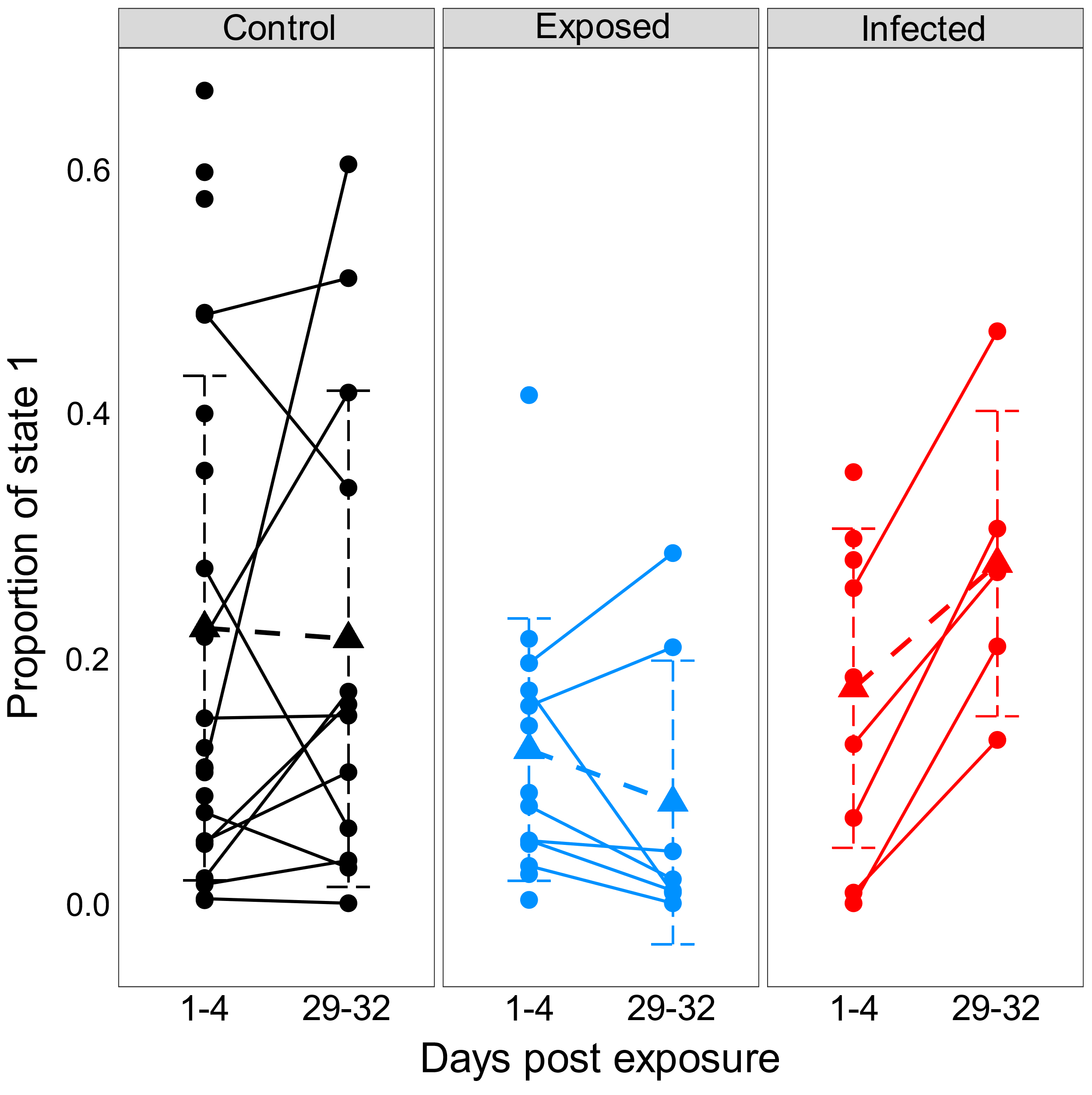


**Supplementary Figure 3:** Individual proportion of time spent in state 1 1-4 and 29-32 dpe. Data points of individuals that were recorded repeatedly are connected by a line. Triangles indicate mean values and are connected by a dashed line. Dashed error bars represent the standard deviation.

**
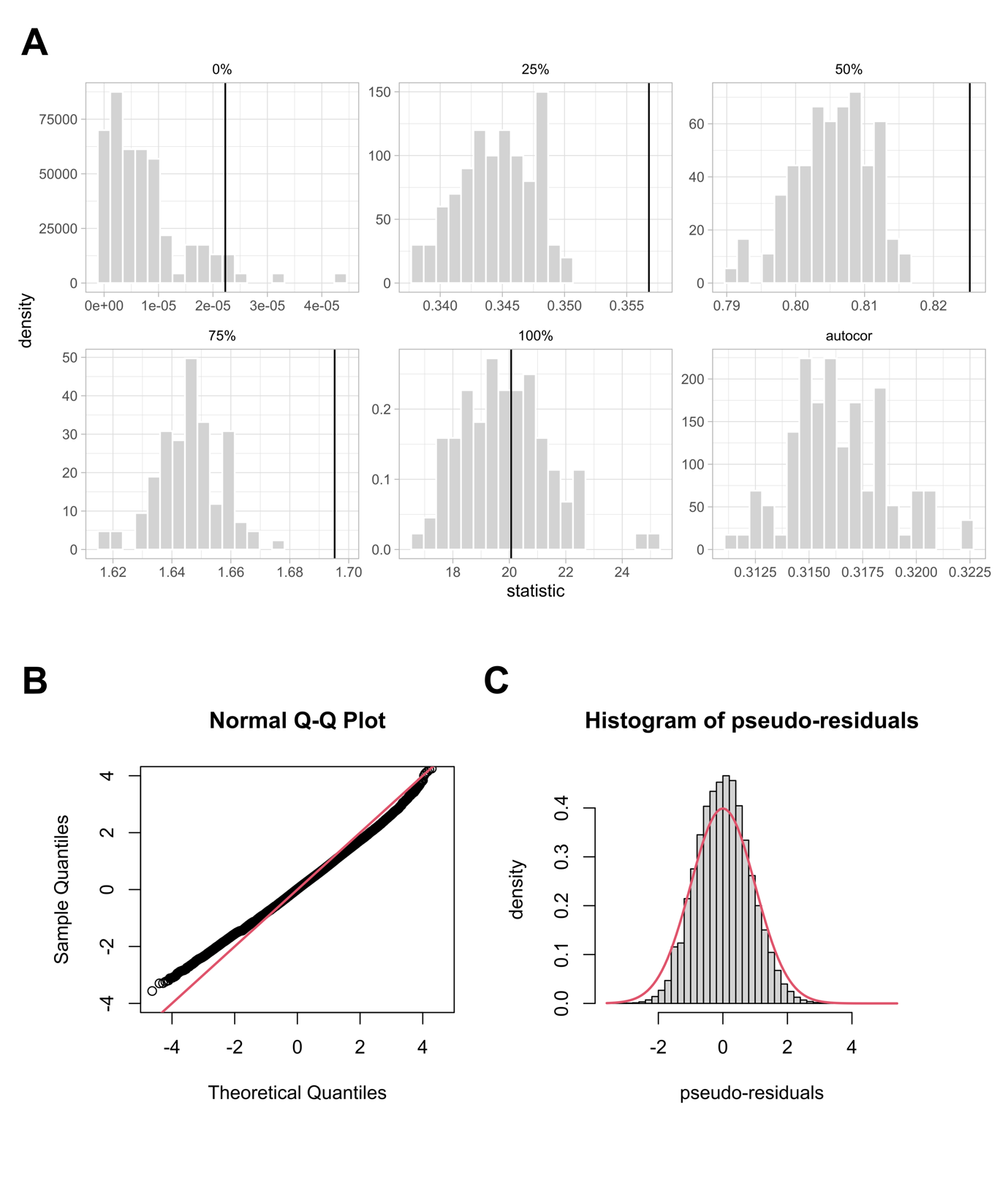
**

**Supplementary Figure 4:** Assessment of goodness-of-model-fit. **(A)** histograms of minimum, maximum, median, and quartiles of simulated observations under the fitted HMM and autocorrelation between consecutive observations based on 100 simulated data sets. The vertical line represents the value observed in the real data. The observed value for the autocorrelation is 0.395 and therefore exceeds the scale of the simulated autocorrelation histogram. **(B)** Normality of pseudo-residuals under the fitted HMM. Sample quantiles (y-axis) are plotted against theoretical quantiles (x-axis) and the red line corresponds to the standard normal distribution. **(C)** Histogram of pseudo-residuals with the red line corresponding to the standard normal distribution.


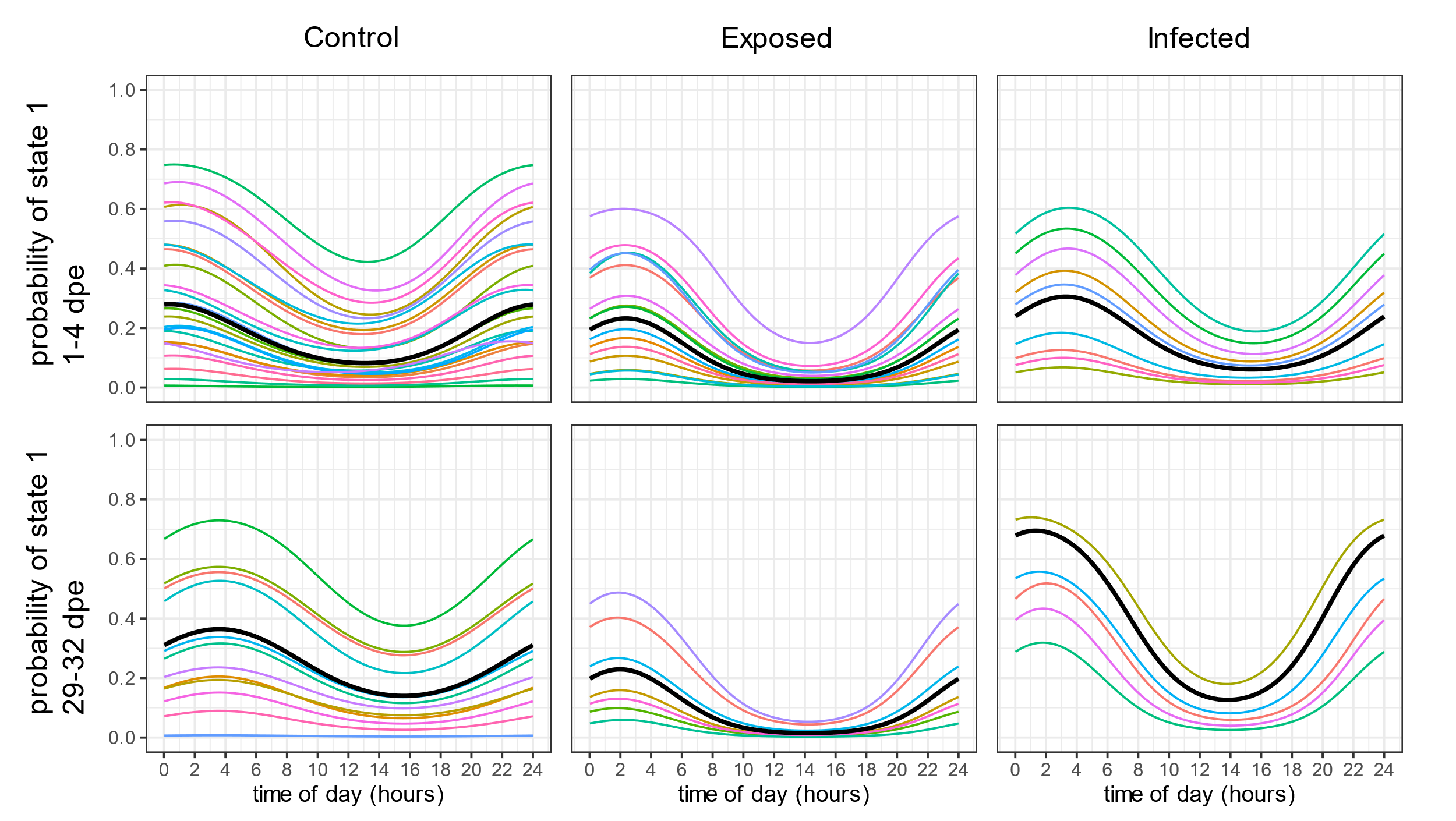


**Supplementary Figure 5:** Heterogeneity in sleep behavior among individuals. Each colored line represents the probability of an individual occupying the sleep state 1, corresponding to the periodic stationary distribution of the HMM, per recording time. The black line indicates the mean for each treatment and recording time.


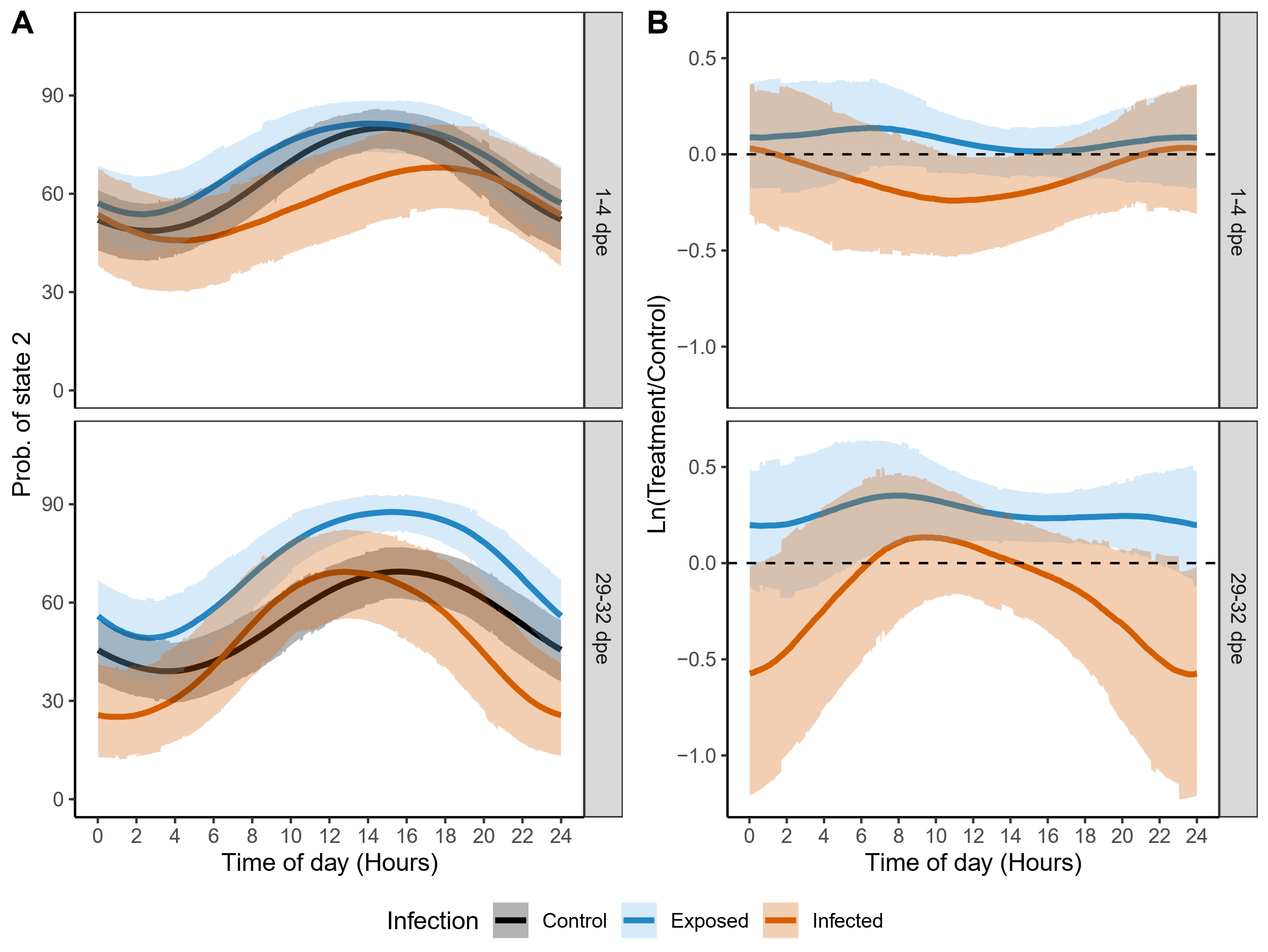


**Supplementary Figure 6. (A)** Probabilities (i.e., expected percentages) for control, exposed and infected fish of occupying state 2 (moderate activity), corresponding to the periodic stationary distribution of the HMM, per recording time (1-4- and 29-32 dpe). Middle lines display the mean probabilities and upper and lower areas the respective 95% confidence intervals. **(B)** Logarithmic ratio of the deviation in state 2 of exposed and infected fish from the respective control (dashed line) derived from simulations based on the HMM. Middle lines display the means and upper and lower areas the 95% confidence intervals of the distribution.


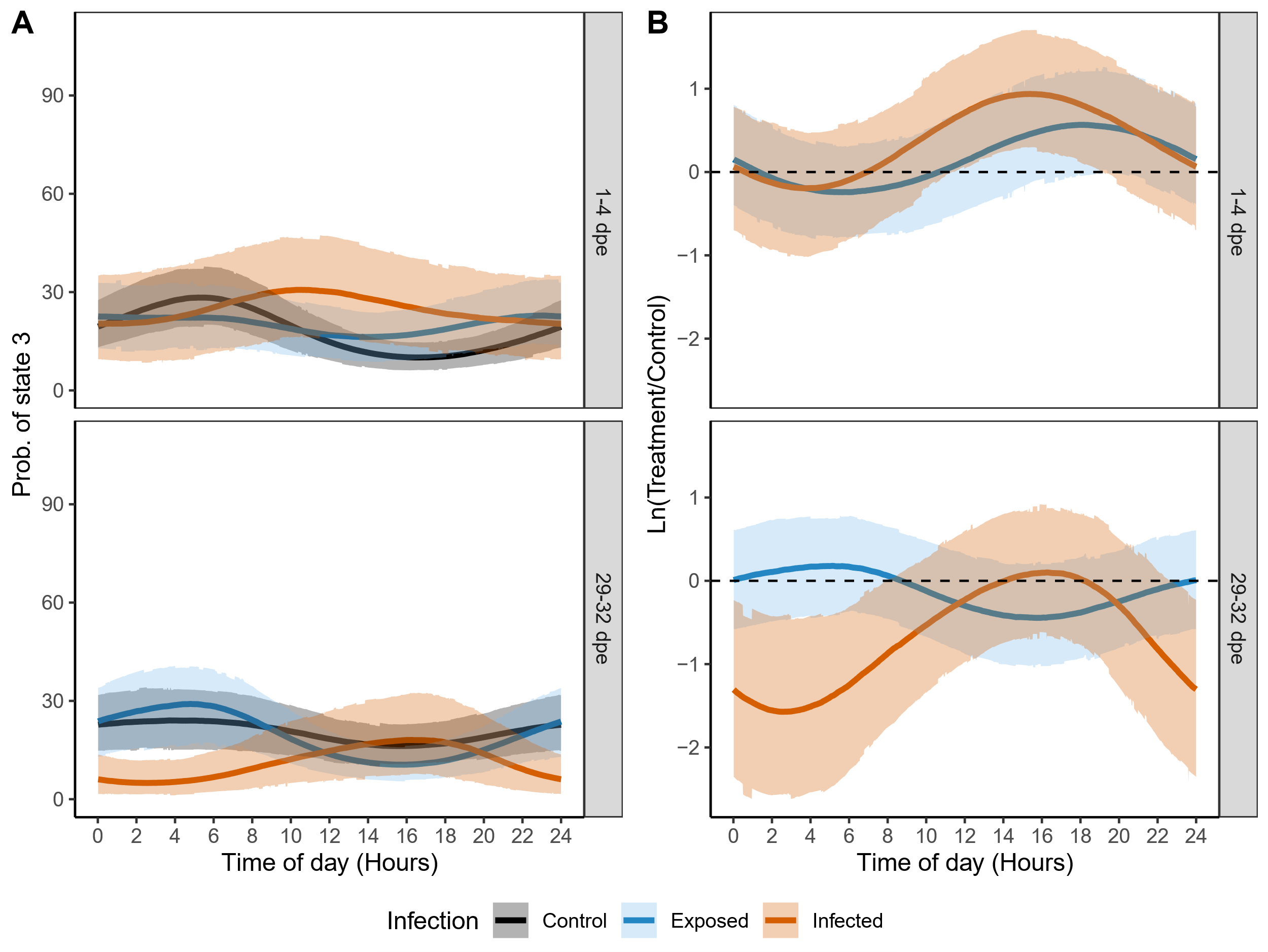


**Supplementary Figure 7. (A)** Probabilities (i.e., expected percentages) for control, exposed and infected fish of occupying state 3 (high activity), corresponding to the periodic stationary distribution of the HMM, per recording time (1-4- and 29-32 dpe). Middle lines display the mean probabilities and upper and lower areas the respective 95% confidence intervals. **(B)** Logarithmic ratio of the deviation in state 3 of exposed and infected fish from the respective control (dashed line) derived from simulations based on the HMM. Middle lines display the means and upper and lower areas the 95% confidence intervals of the distribution.


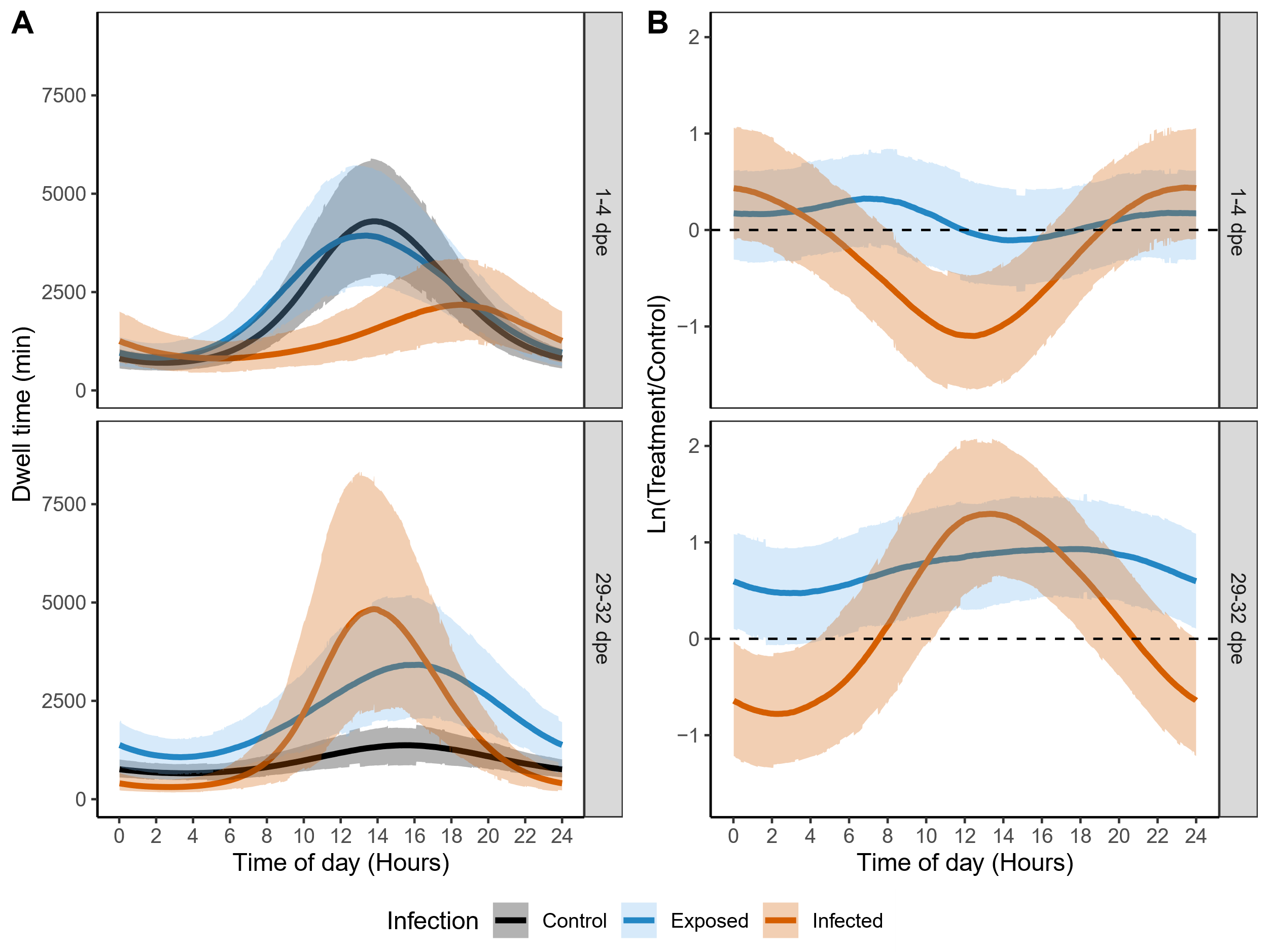


**Supplementary Figure 8. (A)** Expected dwell times (i.e., time spent continuously in one state) as a function of the time of day for control, exposed and infected fish in state 2 (moderate activity) per recording time. Middle lines display the mean dwell times and upper and lower lines the respective 95% confidence intervals. **(B)** Logarithmic ratio of the deviation in state 2 of exposed and infected fish from the respective control (dashed line) derived from simulations based on the HMM. Middle lines display the means and upper and lower areas the 95% confidence intervals of the distribution.


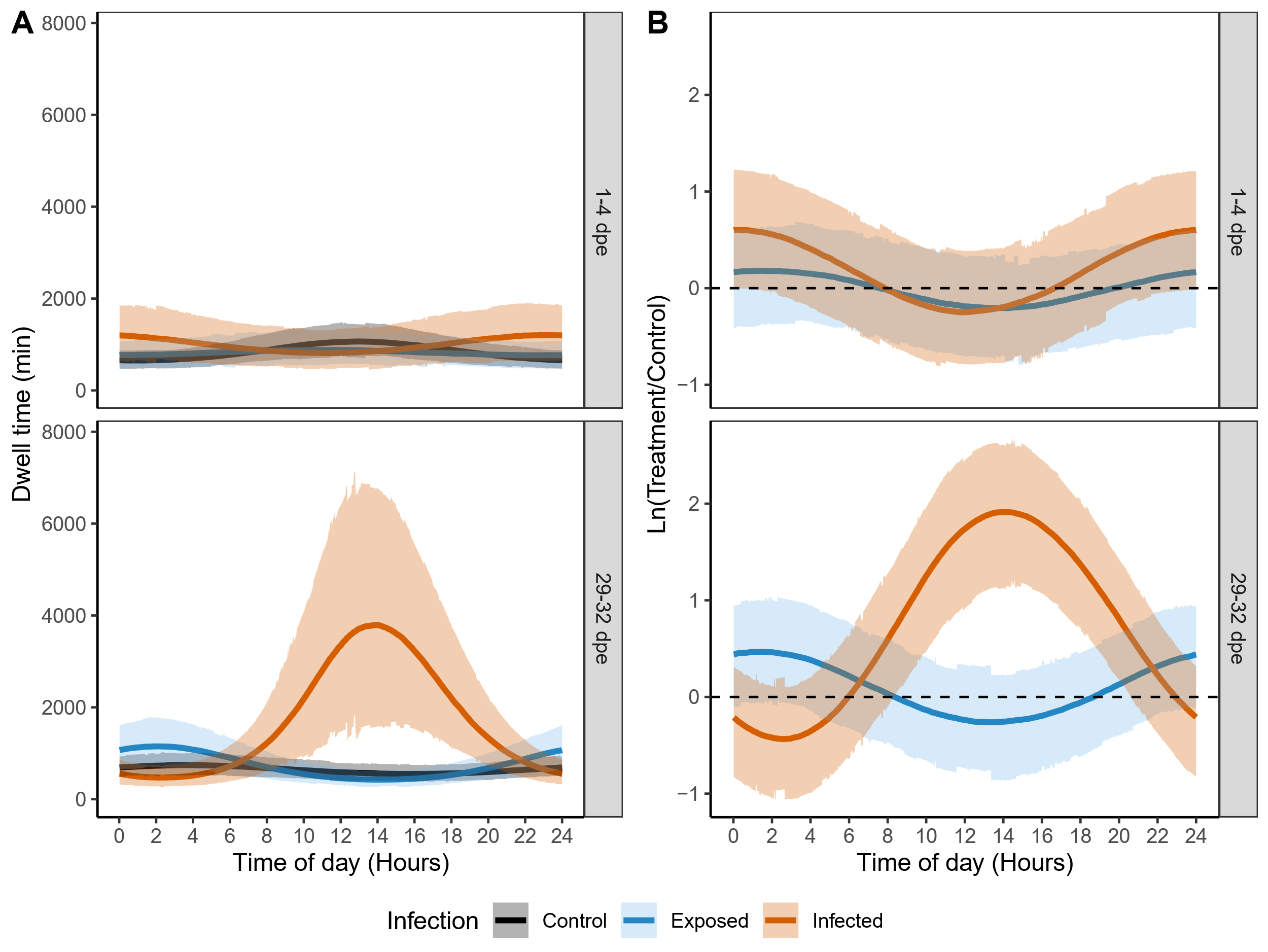


**Supplementary Figure 9. (A)** Expected dwell times (i.e., time spent continuously in one state) as a function of the time of day for control, exposed and infected fish in state 3 (high activity) per recording time. Middle lines display the mean dwell times and upper and lower lines the respective 95% confidence intervals. **(B)** Logarithmic ratio of the deviation in state 3 of exposed and infected fish from the respective control (dashed line) derived from simulations based on the HMM. Middle lines display the means and upper and lower areas the 95% confidence intervals of the distribution.


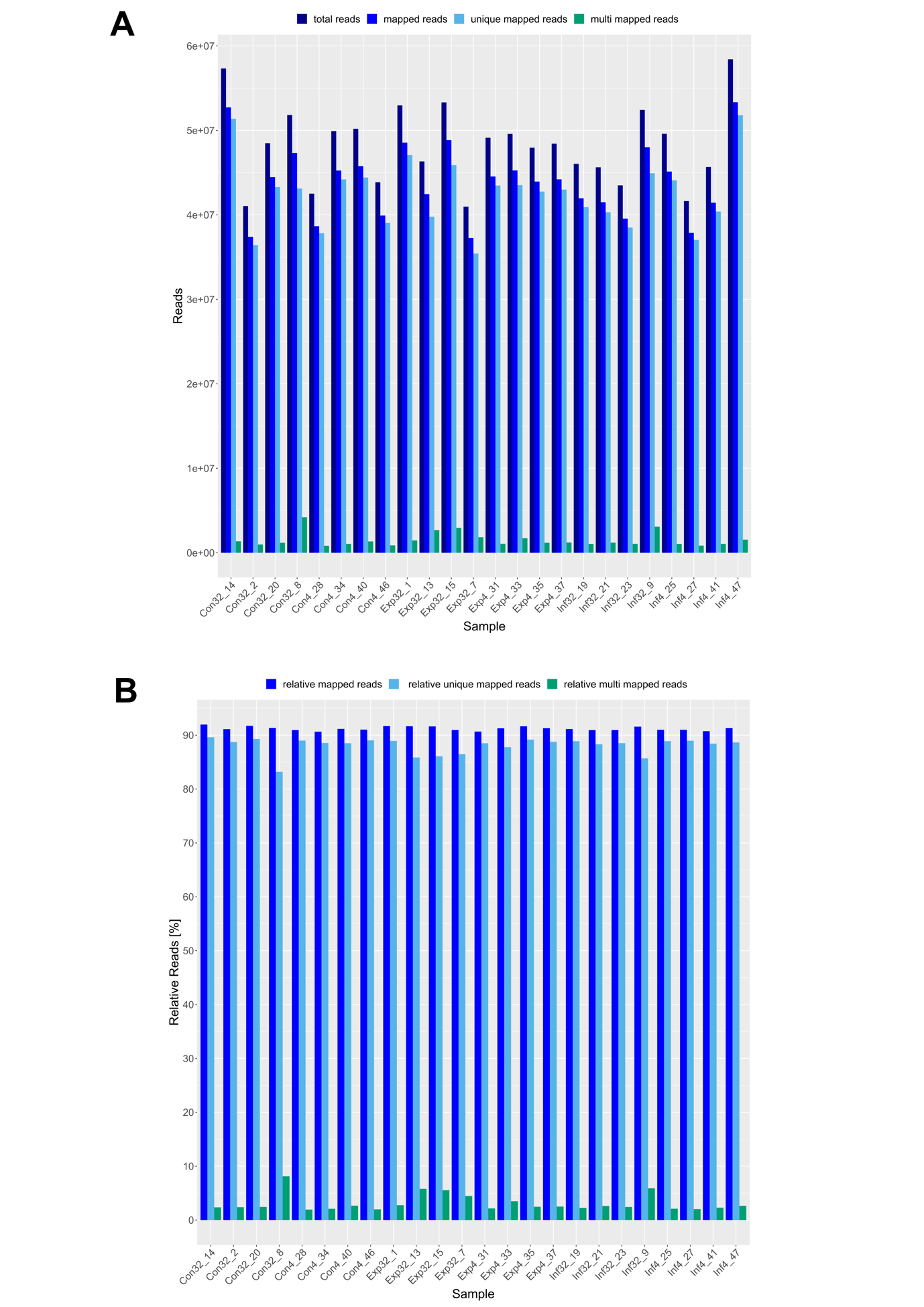


**Supplementary Figure 10: (A)** Total and **(B)** relative number of mapped, uniquely mapped, and multi-mapped reads for all brain samples used for RNA-sequencing. Brain samples originate from control (Con), exposed (Exp), and infected (Inf) individuals 4 and 32 dpe. The number after the underline represents the individual fish ID

**References**

1. Hu J, Yang Z, Li X, Lu H. 2016 C-C motif chemokine ligand 20 regulates neuroinflammation following spinal cord injury via Th17 cell recruitment. *J Neuroinflammation* **13**, 1–14. (doi:10.1186/S12974-016-0630-7/FIGURES/9)

2. Ito T, Carson WF, Cavassani KA, Connett JM, Kunkel SL. 2011 CCR6 as a mediator of immunity in the lung and gut. *Exp Cell Res* **317**, 613–619. (doi:10.1016/J.YEXCR.2010.12.018)

3. Jiang H, Tsang L, Wang H, Liu C. 2021 IFI44L as a Forward Regulator Enhancing Host Antituberculosis Responses. *J Immunol Res* **2021**. (doi:10.1155/2021/5599408)

4. Yan Y, Su J, Zhang Z. 2022 The CXCL12/CXCR4/ACKR3 Response Axis in Chronic Neurodegenerative Disorders of the Central Nervous System: Therapeutic Target and Biomarker. *Cell Mol Neurobiol* **42**, 2147–2156. (doi:10.1007/S10571-021-01115-1/TABLES/1)

5. Monti JM, Jantos H. 2008 Activation of the serotonin 5-HT3 receptor in the dorsal raphe nucleus suppresses REM sleep in the rat. *Prog Neuropsychopharmacol Biol Psychiatry* **32**, 940–947. (doi:10.1016/J.PNPBP.2007.12.024)

6. Staner L, Linker T, Toussaint M, Danjou P, Roegel JC, Luthringer R, Le Fur G, Macher JP. 2001 Effects of the selective activation of 5-HT3 receptors on sleep: a polysomnographic study in healthy volunteers. *European Neuropsychopharmacology* **11**, 301–305. (doi:10.1016/S0924-977X(01)00099-2)

7. Chen F, Lu XJ, Nie L, Ning YJ, Chen J. 2018 Molecular characterization of a CC motif chemokine 19-like gene in ayu (Plecoglossus altivelis) and its role in leukocyte trafficking. *Fish Shellfish Immunol* **72**, 301–308. (doi:10.1016/J.FSI.2017.11.012)

8. Sher S, Green A, Khatib S, Dagan Y. 2021 The Possible Role of Endozepines in Sleep Regulation and Biomarker of Process S of the Borbély Sleep Model. *Chronobiol Int* **38**, 122–128. (doi:10.1080/07420528.2020.1849252)

9. Yan A, Zhang T, Yang X, Shao J, Fu N, Shen F, Fu Y, Xia W. 2016 Thromboxane A2 receptor antagonist SQ29548 reduces ischemic stroke-induced microglia/macrophages activation and enrichment, and ameliorates brain injury. *Scientific Reports 2016 6:1* **6**, 1–13. (doi:10.1038/srep35885)

10. Chang MX, Xiong F, Wu XM, Hu YW. 2021 The expanding and function of NLRC3 or NLRC3-like in teleost fish: Recent advances and novel insights. *Dev Comp Immunol* **114**. (doi:10.1016/J.DCI.2020.103859)

11. Brown TM, Hughes AT, Piggins HD. 2005 Gastrin-Releasing Peptide Promotes Suprachiasmatic Nuclei Cellular Rhythmicity in the Absence of Vasoactive Intestinal Polypeptide-VPAC2 Receptor Signaling. *Journal of Neuroscience* **25**, 11155–11164. (doi:10.1523/JNEUROSCI.3821-05.2005)

12. Van Den Pol AN, Yao Y, Fu LY, Foo K, Huang H, Coppari R, Lowell BB, Broberger C. 2009 Neuromedin B and Gastrin-Releasing Peptide Excite Arcuate Nucleus Neuropeptide Y Neurons in a Novel Transgenic Mouse Expressing Strong Renilla Green Fluorescent Protein in NPY Neurons. *The Journal of Neuroscience* **29**, 4622. (doi:10.1523/JNEUROSCI.3249-08.2009)

13. Chitu V, ¸ölen Gokhan S, Nandi S, Mehler MF, Stanley ER. 2016 Emerging Roles for CSF-1 Receptor and its Ligands in the Nervous System. (doi:10.1016/j.tins.2016.03.005)

14. McAlpine CS *et al.* 2019 Sleep modulates haematopoiesis and protects against atherosclerosis. *Nature* (doi:10.1038/s41586-019-0948-2)

15. Lye E, Mirtsos C, Suzuki N, Suzuki S, Yeh WC. 2004 The role of interleukin 1 receptor-associated kinase-4 (IRAK-4) kinase activity in IRAK-4-mediated signaling. *J Biol Chem* **279**, 40653–40658. (doi:10.1074/JBC.M402666200)

16. Im E, Jung J, Rhee SH. 2012 Toll-Like Receptor 5 Engagement Induces Interleukin-17C Expression in Intestinal Epithelial Cells. *Journal of Interferon & Cytokine Research* **32**, 583. (doi:10.1089/JIR.2012.0053)

17. Zhong Y, Ye Q, Chen C, Wang M, Wang H. 2018 Ezh2 promotes clock function and hematopoiesis independent of histone methyltransferase activity in zebrafish. *Nucleic Acids Res* **46**, 3382. (doi:10.1093/NAR/GKY101)

18. Tai H-H, Cho H, Tong M, Ding Y. 2006 NAD+-Linked 15-Hydroxyprostaglandin Dehydrogenase: Structure and Biological Functions. *Curr Pharm Des* **12**, 955–962. (doi:10.2174/138161206776055958)

19. Pilla-Moffett D, Barber MF, Taylor GA, Coers J. 2016 Interferon-inducible GTPases in host resistance, inflammation and disease. *J Mol Biol* **428**, 3495. (doi:10.1016/J.JMB.2016.04.032)

20. Wang X, Yin G, Zhang W, Song K, Zhang L, Guo Z. 2021 Prostaglandin Reductase 1 as a Potential Therapeutic Target for Cancer Therapy. *Front Pharmacol* **12**. (doi:10.3389/FPHAR.2021.717730)

21. Chacón PJ, del Marco Á, Arévalo Á, Domínguez-Giménez P, García-Segura LM, Rodríguez-Tébar A. 2015 Cerebellin 4, a synaptic protein, enhances inhibitory activity and resistance of neurons to amyloid-β toxicity. *Neurobiol Aging* **36**, 1057–1071. (doi:10.1016/J.NEUROBIOLAGING.2014.11.006)

22. Nebert DW, Vasiliou V. 2004 Analysis of the glutathione S-transferase (GST) gene family. *Hum Genomics* **1**, 460–464. (doi:10.1186/1479-7364-1-6-460/FIGURES/2)

23. Rebeaud F *et al.* 2008 The proteolytic activity of the paracaspase MALT1 is key in T cell activation. *Nature Immunology 2008 9:3* **9**, 272–281. (doi:10.1038/ni1568)

24. Panula P, Aarnisalo AA, Wasowicz K. 1996 Neuropeptide FF, a mammalian neuropeptide with multiple functions. *Prog Neurobiol* **48**, 461–479. (doi:10.1016/0301-0082(96)00001-9)

25. Sun YL, Zhang XY, He N, Sun T, Zhuang Y, Fang Q, Wang KR, Wang R. 2012 Neuropeptide FF activates ERK and NF kappa B signal pathways in differentiated SH-SY5Y cells. *Peptides (N.Y.)* **38**, 110–117. (doi:10.1016/J.PEPTIDES.2012.08.019)

26. Liu X, Cao X, Wang S, Ji G, Zhang S, Li H. 2017 Identification of Ly2 members as antimicrobial peptides from zebrafish Danio rerio. *Biosci Rep* **37**. (doi:10.1042/BSR20160265)

27. Goldstein N, Levine BJ, Loy KA, Duke WL, Meyerson OS, Jamnik AA, Carter ME. 2018 Hypothalamic Neurons that Regulate Feeding Can Influence Sleep/Wake States Based on Homeostatic Need. *Curr Biol* **28**, 3736-3747.e3. (doi:10.1016/J.CUB.2018.09.055)

28. Bédard T, Mountney C, Kent P, Anisman H, Merali Z. 2007 Role of gastrin-releasing peptide and neuromedin B in anxiety and fear-related behavior. *Behavioural Brain Research* **179**, 133–140. (doi:10.1016/J.BBR.2007.01.021)

29. Kagawa Y, Low YL, Pyun J, Doglione U, Short JL, Pan Y, Nicolazzo JA. 2023 Fatty Acid-Binding Protein 4 is Essential for the Inflammatory and Metabolic Response of Microglia to Lipopolysaccharide. *J Neuroimmune Pharmacol* **18**, 448–461. (doi:10.1007/S11481-023-10079-6)

30. Lee JM, Kim H, Baek SH. 2021 Unraveling the physiological roles of retinoic acid receptor-related orphan receptor α. *Experimental & Molecular Medicine 2021 53:9* **53**, 1278–1286. (doi:10.1038/s12276-021-00679-8)

31. Hayashi F *et al.* 2001 The innate immune response to bacterial flagellin is mediated by Toll-like receptor 5. *Nature 2001 410:6832* **410**, 1099–1103. (doi:10.1038/35074106)

32. Guo F *et al.* 2015 Adipocyte-derived PAMM suppresses macrophage inflammation by inhibiting MAPK signalling. *Biochem J* **472**, 309–318. (doi:10.1042/BJ20150019)

33. Wilcox AG, Vizor L, Parsons MJ, Banks G, Nolan PM. 2017 Inducible Knockout of Mouse Zfhx3 Emphasizes Its Key Role in Setting the Pace and Amplitude of the Adult Circadian Clock. *J Biol Rhythms* **32**, 433–443. (doi:10.1177/0748730417722631)

34. Lazarus M, Oishi Y, Bjorness TE, Greene RW. 2019 Gating and the need for sleep: Dissociable effects of adenosine a1and a2areceptors. *Front Neurosci* **13**, 463244. (doi:10.3389/FNINS.2019.00740/BIBTEX)

35. Hanington PC, Belosevic M. 2007 Interleukin-6 family cytokine M17 induces differentiation and nitric oxide response of goldfish (Carassius auratus L.) macrophages. *Dev Comp Immunol* **31**, 817–829. (doi:10.1016/J.DCI.2006.12.001)

36. Opp MR. 2005 Cytokines and sleep. *Sleep Med Rev* **9**, 355–364. (doi:10.1016/J.SMRV.2005.01.002)

37. He Y, Jones CR, Fujiki N, Xu Y, Guo B, Holder JL, Rossner MJ, Nishino S, Fu YH. 2009 The Transcriptional Repressor DEC2 Regulates Sleep Length in Mammals. *Science* **325**, 866. (doi:10.1126/SCIENCE.1174443)

38. Pellegrino R *et al.* 2014 A novel BHLHE41 variant is associated with short sleep and resistance to sleep deprivation in humans. *Sleep* **37**, 1327–1336. (doi:10.5665/SLEEP.3924)
